# Reproducing Five Motor Behaviors in a Salamander Robot With Virtual Muscles and a Distributed CPG Controller Regulated by Drive Signals and Proprioceptive Feedback

**DOI:** 10.3389/fnbot.2020.604426

**Published:** 2020-12-23

**Authors:** Jérémie Knüsel, Alessandro Crespi, Jean-Marie Cabelguen, Auke J. Ijspeert, Dimitri Ryczko

**Affiliations:** ^1^Biorobotics Laboratory (BioRob), Institute of Bioengineering, Ecole Polytechnique Fédérale de Lausanne (EPFL), Lausanne, Switzerland; ^2^Institute for Optimisation and Data Analysis (IODA), Bern University of Applied Sciences, Biel, Switzerland; ^3^Institut National de la Santé et de la Recherche Médicale (INSERM) U 862 - Neurocentre Magendie, Université de Bordeaux, Bordeaux, France; ^4^Département de Pharmacologie-Physiologie, Faculté de Médecine et des Sciences de la Santé, Université de Sherbrooke, Sherbrooke, QC, Canada; ^5^Centre de Recherche du Centre Hospitalier Universitaire de Sherbrooke, Sherbrooke, QC, Canada; ^6^Institut de Pharmacologie de Sherbrooke, Sherbrooke, QC, Canada; ^7^Centre d'Excellence en Neurosciences de l'Université de Sherbrooke, Sherbrooke, QC, Canada

**Keywords:** central pattern generator (CPG), proprioceptive sensory feedback, descending drive, distributed control, salamander, locomotion, numerical modeling, robotics

## Abstract

Diverse locomotor behaviors emerge from the interactions between the spinal central pattern generator (CPG), descending brain signals and sensory feedback. Salamander motor behaviors include swimming, struggling, forward underwater stepping, and forward and backward terrestrial stepping. Electromyographic and kinematic recordings of the trunk show that each of these five behaviors is characterized by specific patterns of muscle activation and body curvature. Electrophysiological recordings in isolated spinal cords show even more diverse patterns of activity. Using numerical modeling and robotics, we explored the mechanisms through which descending brain signals and proprioceptive feedback could take advantage of the flexibility of the spinal CPG to generate different motor patterns. Adapting a previous CPG model based on abstract oscillators, we propose a model that reproduces the features of spinal cord recordings: the diversity of motor patterns, the correlation between phase lags and cycle frequencies, and the spontaneous switches between slow and fast rhythms. The five salamander behaviors were reproduced by connecting the CPG model to a mechanical simulation of the salamander with virtual muscles and local proprioceptive feedback. The main results were validated on a robot. A distributed controller was used to obtain the fast control loops necessary for implementing the virtual muscles. The distributed control is demonstrated in an experiment where the robot splits into multiple functional parts. The five salamander behaviors were emulated by regulating the CPG with two descending drives. Reproducing the kinematics of backward stepping and struggling however required stronger muscle contractions. The passive oscillations observed in the salamander's tail during forward underwater stepping could be reproduced using a third descending drive of zero to the tail oscillators. This reduced the drag on the body in our hydrodynamic simulation. We explored the effect of local proprioceptive feedback during swimming and forward terrestrial stepping. We found that feedback could replace or reduce the need for different drives in both cases. It also reduced the variability of intersegmental phase lags toward values appropriate for locomotion. Our work suggests that different motor behaviors do not require different CPG circuits: a single circuit can produce various behaviors when modulated by descending drive and sensory feedback.

## Introduction

Many motor behaviors in animals require coordinated rhythmic activation of multiple muscles. Neural networks capable of producing such activity patterns without rhythmic input from other networks or from sensory feedback are called central pattern generators (CPGs). It has been shown that CPGs in the spinal cord underlie locomotion in many vertebrate species (for review, Grillner and El Manira, 2020). Drive signals descending from brain neurons control locomotion initiation, speed and gait transitions (Brocard et al., 2010; Capelli et al., 2017; Caggiano et al., 2018; Josset et al., 2018) and steering movements (Fagerstedt et al., 2001; Ryczko et al., 2016b; Cregg et al., 2020). Sensory feedback plays an important role in modulating the CPG activity to adapt the locomotor pattern to the environment (e.g., Wyart et al., 2009; Akay et al., 2014; Hubbard et al., 2016; Knafo et al., 2017). These feedback signals depend on the interactions between the neural networks, the mechanical properties of the body and the environment, making it a challenge to fully understand the operation of the CPG even at a high level of abstraction. Numerical models of the complete system can be used to investigate the effect of sensory feedback on the CPG, but some aspects such as hydrodynamic and friction forces are difficult to simulate reliably. Robots are thus useful to validate simulation results in the real world, with real physics. Here, we used numerical simulations and robotics to investigate the generation of different behaviors in the salamander, an interesting animal model as it can move underwater and on ground (Ryczko et al., 2020). In particular, we addressed the following questions:

1. Can different motor behaviors be generated by a single spinal CPG circuit as opposed to requiring several dedicated CPG circuits?
2. What are the roles of descending drives in generating these different motor behaviors, and how many independent drives are necessary?
3. What is the potential role of sensory feedback in shaping the patterns and in reducing the variability of CPG activity observed in isolated spinalcords?

We used the *Salamandra robotica II* robot (Crespi et al., 2013) driven by a spinal CPG model and virtual muscles to reproduce the five salamander behaviors documented in Ryczko et al. (2015): forward swimming, forward and backward terrestrial stepping, forward underwater stepping, and struggling. To match the biological data from that study, we focused on reproducing the patterns of muscle activation and body curvature along the body axis.

For the CPG, our starting point was the abstract oscillator model of Ijspeert et al. (2007), with modifications to allow for the flexible coordination of limb and axial network activities (Knüsel et al., 2013). This flexibility is required to reproduce the observations from Ryczko et al. (2015). Here, we extended the model to comprise 25 segments and introduced random parameters to account for the differences between individuals. The main hypotheses are the following: (1) limb oscillators project only to the axial oscillators close to the corresponding girdles; (2) couplings between axial oscillators are stronger in the head-to-tail direction; (3) limb oscillators saturate^1^ at lower excitatory drives than axial oscillators; (4) hindlimb oscillators are intrinsically slower than forelimb oscillators. Hypotheses 1 and 2 make the model's intersegmental phase lag flexible and controllable (Knüsel et al., 2013). Hypotheses 3 and 4 allow the model to reproduce the distribution of phase lags of recordings *in vitro*.

We modeled the biomechanical properties of the body axis using virtual muscles that determine the torques of the axial joints based on the CPG activity and the current joint position and velocity (Ekeberg, 1993). The joint positions were also used for proprioceptive feedback, simulating stretch receptors that send phasic inputs to the local CPG segments. The virtual muscle model requires a small time step for stability and accuracy of the numerical integration, which is challenging to achieve with eight joints given the limited bandwidth and processing power of the robotic platform. We solved this difficulty by distributing the computation of the CPG and muscle models in the eight active modules, so that each module calculates the part of the model that controls its own joint. The modules use peer-to-peer communication, such that splitting the robot results in several functional pieces (unlike most robots).

The isolated CPG model was tuned to reproduce the diversity of coordination patterns observed in isolated spinal cords. To determine values for the proprioceptive feedback and virtual muscle parameters, we systematically explored the parameter space using a mechanical simulation of the robot: Using the same tonic drive for all oscillators, we identified parameter values that allowed feedback to have a positive effect on swimming speed while maintaining stable CPG rhythms. We then attempted to reproduce the five salamander behaviors without sensory feedback, by varying the tonic drive sent to different parts of the CPG, adapting other model parameters when necessary. Further simulations were made to investigate the effect of proprioceptive feedback during swimming and forward terrestrial stepping. Finally, we used the real robot to validate simulation results. Each behavior was reproduced on the robot using different “individuals” from the family of models used in modeling isolated spinal cords, to check the robustness of the control architecture to individual variations.

## Motor Control in Salamanders

The salamander spinal CPG produces the rhythmic movements of the limbs (Cheng et al., 1998; Lavrov and Cheng, 2004; Ijspeert et al., 2007), trunk (Delvolvé et al., 1999; Branchereau et al., 2000; Ryczko et al., 2010), and tail (Charrier and Cabelguen, 2013). This spinal circuitry is controlled by the brainstem in salamanders as in other vertebrates (for review, see Ryczko and Dubuc, 2013). Stimulation of the salamander mesencephalic locomotor region elicits stepping at low stimulation intensities, whereas swimming requires higher intensities (Cabelguen et al., 2003). These descending commands are carried to the spinal cord by reticulospinal neurons (Ryczko et al., 2016a, see also Ryczko et al., 2020 for a recent review).

The coordination of muscles along the body axis plays an important role in salamander locomotion, to generate thrust during swimming and to maximize the stride length during terrestrial stepping (Delvolvé et al., 1997). So far, at least five salamander motor behaviors have been characterized: forward swimming, forward and backward terrestrial stepping, forward underwater stepping, and struggling (Ryczko et al., 2015). Forward terrestrial stepping generally takes the form of a walking trot, but lateral sequence walks have also been observed (reviewed in Chevallier et al., 2008, see also Ashley-Ross et al., 2009). During forward underwater stepping, the salamander progresses at the bottom of water, with periods of suspension in water without ground contact. Struggling refers to the behavior of the salamander when it is firmly grasped at the pelvic girdle. Electromyographic (EMG) recordings of multiple segments in the salamander mid-trunk show that each of the five behaviors is characterized by a specific pattern of muscle activation, in terms of cycle frequencies and intersegmental phase lags: (1) rostrocaudal waves occur during forward swimming and, with lower cycle frequencies, during backward terrestrial stepping; (2) slow caudorostral waves occur during struggling; (3) standing waves are stable during forward terrestrial stepping but more variable during forward underwater stepping (Ryczko et al., 2015).

Kinematic recordings show similar patterns of trunk curvature. However, kinematic intersegmental phase lags are significantly larger during forward terrestrial stepping and swimming (Frolich and Biewener, 1992; Ryczko et al., 2015). In other words, the delay between muscle activation and body bending gets larger toward the tail. This suggests that the mechanical properties of body tissues play an important role during these behaviors, as suggested by a lamprey modeling study (Tytell et al., 2010).

The increasing EMG-mechanical delay toward the tail also suggests that proprioceptive feedback might have a different effect at various points along the body axis. Salamanders are known to have sensory cells that generate proprioceptive information relative to axial movements: The skin contains mechano-sensitive Merkel cells (Scott et al., 1981; Diamond et al., 1986), and some cells in the spinal cord are morphologically similar to the mechano-sensitive “edge cells” (Schroeder and Egar, 1990) that encode body bending in lampreys (Grillner et al., 1982, 1984). They also have cerebrospinal fluid contacting neurons (Kolmer-Agduhr cells, Harper and Roberts, 1993), which are active during body bending in zebrafish (Böhm et al., 2016) and provide mechanosensory input to the swimming CPG (Wyart et al., 2009; Hubbard et al., 2016, Orts-Del'Immagine et al., 2020, see also Jalalvand et al., 2016 in lampreys). The limbs are another source of proprioceptive feedback, as they contain fibers that respond to stretch similarly to muscle spindles in other species (Bone et al., 1976).

According to *in vitro* recordings of the salamander spinal cord, the isolated CPG can generate stable patterns for the three types of axial waves (caudorostral, standing and rostrocaudal waves), with occasional switches between two wave types (Ryczko et al., 2015). The intersegmental phase lags generated by the isolated CPG cover a greater range than those observed in EMG recording (−12.6 to +12.4% of a cycle duration for recordings *in vitro*, and −4.8 to +6.4% for EMG recordings), with a distribution showing three peaks centered on −9.6, −1.0, and +6.6%. The salamander CPG thus provides a flexible ground onto which sensory feedback and descending drives could act to influence the spinal motor output.

## Related Modeling Work

Previous studies have modeled the CPG components using abstract oscillators (Ijspeert et al., 2005, 2007; Knüsel et al., 2013; Yin et al., 2016), single bursting neurons (Liu et al., 2018, 2020), integrate-and-fire neurons (Ijspeert, 2001; Bem et al., 2003; Harischandra et al., 2011; Knüsel et al., 2013) and detailed networks of three compartment Hodgkin-Huxley neurons (Bicanski et al., 2013).

The mechanical body of the salamander has been modeled with varying accuracy. Many models include four joints between the girdles and a single degree of freedom (DOF) per limb (Ijspeert, 2001; Ijspeert et al., 2005, 2007; Suzuki et al., 2019a) or three DOFs per limb (Harischandra et al., 2010, 2011; Liu et al., 2018, 2020). The simplest model has one of each (Yin et al., 2016), while other models have one joint between the girdles and two DOFs per limb (Zhong et al., 2018; Suzuki et al., 2019b). Bem et al. (2003) have modeled the swimming salamander as a chain of ten links, corresponding roughly to three trunk joints and no limbs. The most accurate model has five joints between the girdles and four DOFs in each limb (Karakasiliotis et al., 2016; Horvat and Ijspeert, 2017; Horvat et al., 2017). Mechanical properties (damping and elasticity) of the body tissues were included in the muscle models used by Ijspeert (2001; 2005) Bem et al. (2003), Harischandra et al. (2010, 2011), and Liu et al. (2018, 2020), and in the controller of Suzuki et al. (2019b).

The effect of sensory feedback on the activity of the salamander CPG has only been investigated in simulation (Bem et al., 2003; Ijspeert et al., 2005; Harischandra et al., 2011; Liu et al., 2020). The role of sensory feedback in body-limb coordination has also been investigated using controllers without CPG, both in simulations (Horvat and Ijspeert, 2017) and with a robot (Suzuki et al., 2019a).

Most studies have focused on the reproduction of forward terrestrial stepping (with a walking and/or trotting gait), swimming, transitions between these behaviors, and turning. The exceptions are the works of Karakasiliotis et al. (2016) which reproduced underwater stepping in addition to swimming and forward terrestrial stepping (though using predefined joint trajectories rather than a CPG) and Liu et al. (2018) which reproduced backward terrestrial stepping in addition to forward terrestrial stepping (using dedicated networks for each gait).

Table 1 summarizes the particularities of past studies and how they compare to the present one. To our knowledge, the present work is the first to incorporate biomechanical properties and proprioceptive sensory feedback in a real salamander robot.

**Table 1:** Related studies.

	**CPG**	**Trunk joints**	**Limb DOFs**	**Robot**	**Biomech**	**Proprio. feedback**		**Force feedback**		**Behaviors**						**Turning**	**Transition**
						**Trunk**	**Limbs**	**Trunk**	**Limbs**	**Swim**	**Walk**	**Trot**	**Back step**	**U.w. step**	**Struggle**		
Bem et al. (2003)	IF	**•••**															
Bicanski et al. (2013)	HH																
Knüsel et al. (2013)	AO+IF																
Ijspeert (2001)	IF	**••••**	**•**														
Ijspeert et al. (2005)	AO	**••••**	**•**														
Ijspeert et al. (2007)	AO	**••••**	**•**														
Harischandra et al. (2010)		**••••**	**•••**														
Harischandra et al. (2011)	IF	**••••**	**•••**														
Yin et al. (2016)	AO	**•**	**•**														
Karakasiliotis et al. (2016)		**•••••**	**••••**														
Horvat et al. (2017)		**•••••**	**••••**														
Horvat and Ijspeert (2017)		**•••••**	**••••**														
Liu et al. (2018)	BN	**••••**	**•••**														
Zhong et al. (2018)		**•**	**••**														
Suzuki et al. (2019a)		**••••**	**•**														
Suzuki et al. (2019b)	AO	**•**	**••**														
Liu et al. (2020)	BN	**••••**	**•••**														
This study	AO	**••••**	**•**														

*CPG: IF, Integrate and fire; HH, Hodgkin-Huxley; AO, Abstract oscillators; BN, Bursting neurons. Trunk joints: counting joints between girdles (a joint on the girdle counts as a half). DOFs: degrees of freedom. The number of black dots represents the number of trunk joints and limb degrees of freedom respectively. Biomech: mechanical properties from body tissues such as muscles. Walk: forward terrestrial stepping with lateral sequence walking. Trot: forward terrestrial stepping with walking trot. Colors indicate different groups of model features: mechanical model (green), sensory feedback modalities (orange), behaviors (gray)*.

## Materials and Methods

### CPG Model

**Figure 1:**
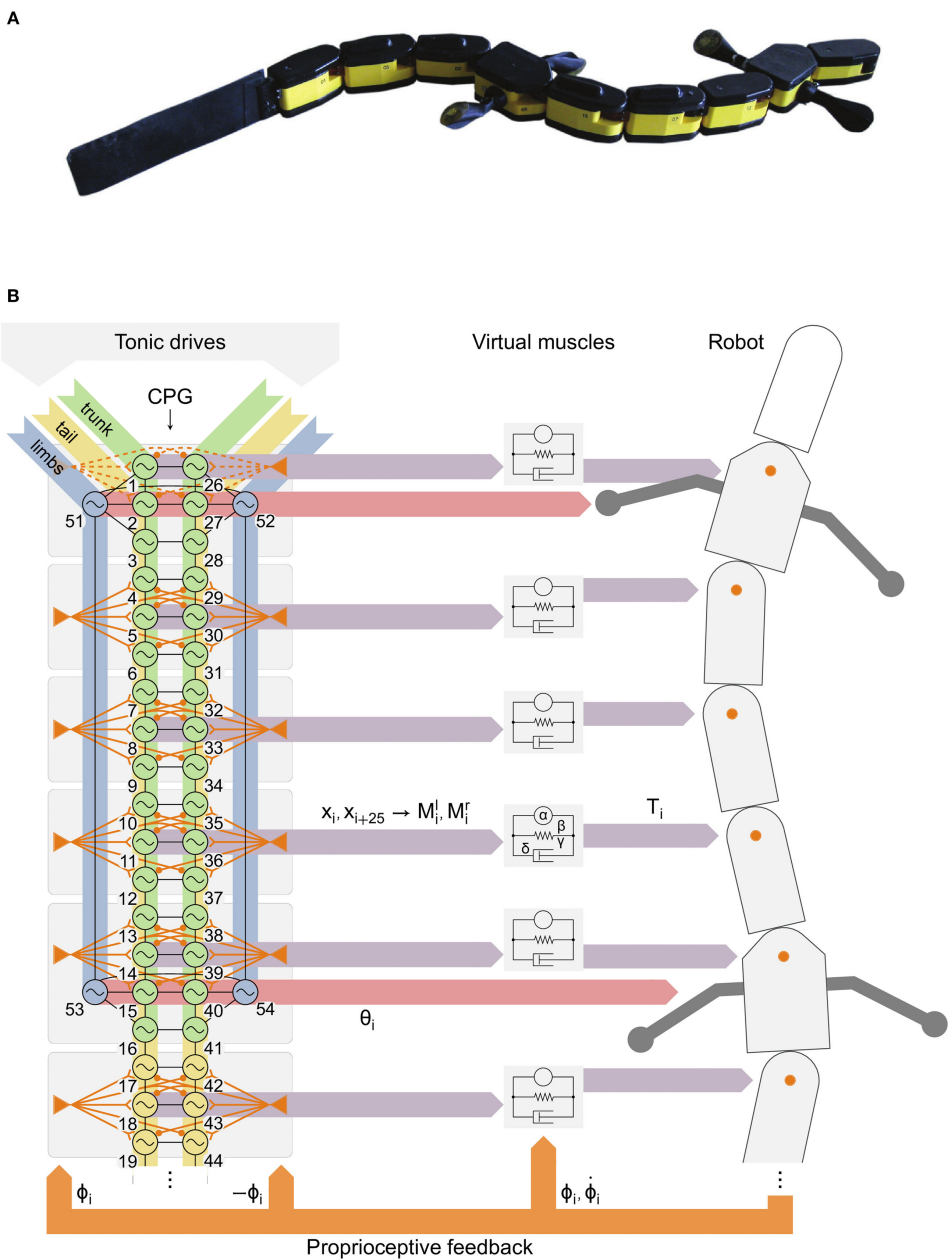
Robot with distributed controller with the spinal central pattern generator (CPG) model, axial proprioceptive feedback, descending drives and virtual muscles. **(A)** The robot *Salamandra robotica II*. **(B)** The axial CPG was divided in 8 groups (gray rectangles) to distribute the computations in the 8 robot modules with active joints. *Left:* tonic descending drives are applied to limb (blue), trunk (green) and tail (yellow) oscillators. Virtual stretch receptors (orange triangles) project to the 3 nearest segments with opposite ipsilateral (excitatory) and contralateral (inhibitory) weights. Feedback from the neck joint (dashed orange) was disabled for robot experiments (see Results). Black lines indicate bidirectional couplings between oscillators (see Figure 2A). *Middle*: 2 outputs *x*_*i*_*, x*_*i*+25_ of each group (purple horizontal arrows) govern left (*l*) and right (*r*) muscle activities *M*_*i*_ from which the muscle model calculates an output torque *T*_*i*_. *Right:* the torque *T*_*i*_ is applied at each axial joint (orange circles). The joint position ϕ_*i*_ and velocity ${\dot{\phi }}_{i}$ are fed back (orange arrows) to the muscles. Virtual stretch receptors only receive ϕ_*i*_. The phases θ_*i*_ of limb oscillators (red horizontal arrows) determine the limb positions.

The model was developed using the Codyn framework and exported to C code to run on the robot microcontrollers. Only the 25 most rostral axial segments (each comprising 1 left and 1 right hemisegments) are modeled out of the 40 segments that salamanders typically have (for review see, Chevallier et al., 2008). Four additional oscillators control the limbs. The 25 axial segments control the active part of the robot. The caudal half of the robot tail is a passive, flexible caudal fin (Figure 1A). Each axial hemisegmental oscillator and each limb oscillator is modeled as a phase oscillator with controllable amplitude, and the connections between oscillators are functions of the phase difference between sender and receiver:

$$\begin{array}{l} {\dot{\theta }}_{i}=2\pi \nu_{i}+\underset{j}{\sum }r_{j}w_{ij}\sin\left(\theta_{j}-\theta_{i}-\varphi_{ij}\right)-\frac{s_{i}}{r_{i}}\sin\theta_{i}\\ {ṙ}_{i}=a\left(R_{i}-r_{i}\right)+s_{i}\cos\theta_{i}\\ x_{i}=r_{i}\left(1+\cos\theta_{i}\right)\\ \nu_{i}=d_{i}e_{i}\\ R_{i}=d_{i}P\left(d_{i},d_{i}^{th}\right) \end{array}$$

A positive output *x*_*i*_ (which determines the muscle activation) is calculated from the instantaneous phase θ_*i*_ and amplitude *r*_*i*_. The intrinsic frequency ν_*i*_ is proportional to the oscillator excitability *e*_*i*_ and to a drive *d*_*i*_ that represents the excitation from descending drives. The intrinsic amplitude *R*_*i*_ increases with increasing drive until it approaches a saturation threshold $d_{i}^{th}$ after which it decreases progressively to zero due to the sigmoid function $P\left(d,d^{th}\right)=\frac{1}{1+e^{b\left(d-d^{th}\right)}}$ with *b* the saturation rate. The excitability *e*_*i*_ determines the intrinsic frequency of a particular oscillator as a function of the external drive. The excitability of each oscillator is drawn from a Gaussian distribution with different means for forelimb, hindlimb and axial oscillators. The saturation thresholds of the forelimbs, hindlimbs and axial network are also drawn from a Gaussian distribution with different means for the axial and limb networks. The coupling from oscillator j to oscillator i is characterized by a strength *w*_*ij*_ and phase bias φ_*ij*_. The gain *a* determines the speed of convergence for the amplitude. The symbol *s*_*i*_ represents the feedback signal from simulated stretch receptors (see below). The terms $-\frac{s_{i}}{r_{i}}\sin\theta_{i}$ and *s*_*i*_cosθ_*i*_ are the polar coordinate equivalent of adding *s*_*i*_ to the derivative ẋ of an oscillator in Cartesian coordinates (see Supplementary Materials for the derivation).

**Figure 2:**
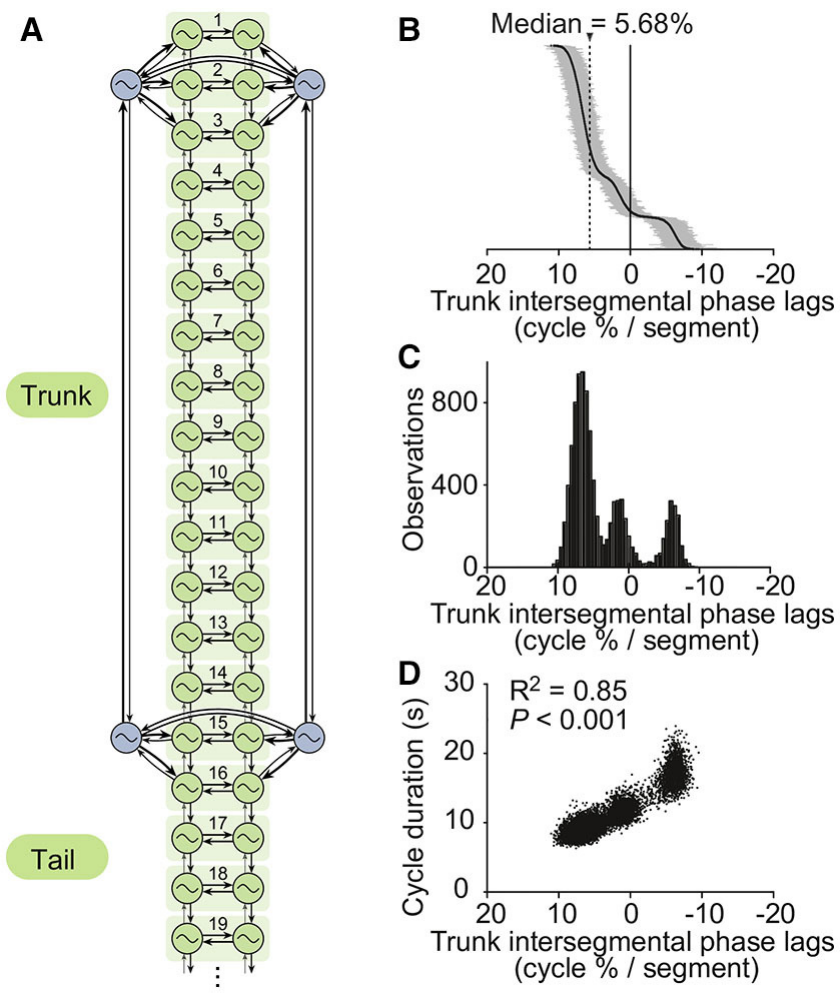
The CPG model. **(A)** The axial (i.e., trunk and tail) spinal network model is constituted by a double chain of 50 oscillators, i.e., 25 segments of which 19 are shown (green). Four oscillators (blue) control the limbs. Thicker arrows denote stronger couplings. For simulations of the isolated CPG, a randomly fluctuating tonic drive mimicking the pharmacological activation used in Ryczko et al. (2015) was applied to all oscillators. **(B)** Intersegmental phase lags from 10,000 simulations of the isolated CPG using different random seeds (intersegmental phase lag calculated by taking the average of intersegmental phase lags between segments 8–12, see Methods). Simulations are ordered by decreasing intersegmental phase lag on the vertical axis. A positive phase lag corresponds to a rostrocaudal traveling wave (i.e., from head to tail), a zero phase lag to a standing wave, and a negative phase lag to a caudorostral traveling wave. **(C)** Trimodal distribution of intersegmental phase lags. **(D)** Cycle durations vs. intersegmental phase lags. A linear fit was applied to the dataset. The square of the correlation coefficient and the significance of the fit are given.

The network connectivity is described in Figure 2A and Table 2. Other parameter values are provided in Tables 3, 4.

**Table 2:** CPG coupling parameters.

**Coupling type**	**Strength *w_***ij***_***	**Phase bias *φ_*ij*_* (rad.)**
Intersegmental, rostrocaudal	5	0.066·2π
Intersegmental, caudorostral	1	−0.066·2π
Intrasegmental, lateral	10	π
Interlimb, rostrocaudal	3	π
Interlimb, caudorostral	30	π
Interlimb, lateral	10	π
Limb to axial oscillators	30	4 (BTS: 5.5^*^)
Axial to limb oscillators	2.5	−4 (BTS: −5.5^*^)

* *BTS, backward terrestrial stepping*.

**Table 3:** Other CPG parameters.

**Name**	**Symbol**	**Value (mean ± SD)**
Amplitude convergence factor	*a*	5
Saturation rate	*b*	500
Drive random walk convergence factor	*c*	0.001 (*in vitro*) 0 (*in vivo*)
Drive random walk step size	σ	0.03 (*in vitro*) 0 (*in vivo*)
Drive	*d_*i*_*	See Table 4
Saturation threshold	$d_{i}^{th}$	0.3 (axis, *in vitro*) 3 (axis, *in vivo*) 0.09 ± 0.02 (limbs, *in vitro*) 1.27 ± 0.02 (limbs, *in vivo*)
Excitability	*e_*i*_*	1.1 ± 0.07 (axis) 0.8 ± 0.05 (forelimbs) 0.5 ± 0.03 (hindlimbs)
Axial proprioceptive feedback, ipsilateral	*w^*ipsi*^*	See Table 4
Axial proprioceptive feedback, contralateral	*w^*contra*^*	*-w^*ipsi*^*

**Table 4:** Parameters regulating the CPG activity.

	**Drives** ***d**_*****i*****_*			**Feedback weights**	
	**Seg. 1–3**	**Seg. 4–25**	**Limbs**	***w^***ipsi***^***	***w^***limb***^***
**Robot experiments with 5 individuals (****Figure 5****)**					
Swimming	1			10	0
Forward terrestrial stepping	0.60 ± 0.02		1.00 ± 0.04	0	0
Forward underwater stepping	0.42 ± 0.01		0.71 ± 0.03	0	0
Backward terrestrial stepping	0.23 ± 0.01		0.46 ± 0.02	0	0
Struggling	0.27 ± 0.01		0.38 ± 0.01	0	0
**Other robot experiments**					
Swimming without regulation (Supplementary Movie 1)	1			0	0
Swimming with differential drive (Supplementary Movie 2)	0.9	1		0	0
Forward terrestrial stepping with feedback (Figure 6E)	0.61		0.98	from −10 to 6	0
**Simulations**					
Isolated CPG (simulation of *in vitro* experiments) (Figures 2, 3)	0.1 ± 0.01			0	0
Swimming without regulation (Supplementary Figure 4A)	1.34			0	0
Swimming with differential drive (Supplementary Figure 4B)	1.03	1.34		0	0
Swimming with axial feedback (Supplementary Figures 4C,D)	1.34			21	0
Forward terrestrial stepping without regulation (Figure 6A)	0.98			0	0
Forward terrestrial stepping with differential drive (Figure 6B)	0.63		0.98	0	0
Forward terrestrial stepping with axial feedback (Figure 6C)	0.98			−0.65	0
Forward terrestrial stepping with limb feedback (Figure 6D)	0.98			0	3.7

*Robot experiments used lower drives for swimming to stay in the robot operating range (see Results). Standard deviations for the drive reflect variations between the simulated individuals. Drive values are shown centered across two or three columns in cases where the same value was applied to the corresponding groups of oscillators*.

### Simulations of the Isolated CPG

For simulations of *in vitro* electrophysiological recordings of the isolated salamander spinal cord reported in Ryczko et al. (2015), the same drive *d*_*i*_ = *d* was used for all oscillators to represent a tonic pharmacological stimulation, with small fluctuations over time added in the form of a mean reverting random walk: $\dot{d}$ = *c*(*d*_0_ − *d*) ± σ with *d*_0_ the drive picked from a Gaussian distribution, *c* a convergence factor, and ±σ a random process yielding positive and negative steps with equal probability. Multiple simulations were performed with different random seeds to reflect the diversity of coordination patterns observed in individual spinal cord preparations.

### Muscle Model

A linear spring-damper model with variable stiffness (Ekeberg, 1993) was used to model a pair of antagonist muscles and calculate the resulting torque at each axial joint (Figure 1B):

$$\begin{array}{l} T_{i}=\alpha \left(M_{i}^{l}-M_{i}^{r}\right)-\beta \left(M_{i}^{l}+M_{i}^{r}+\gamma \right)\phi_{i}-\delta {\dot{\phi }}_{i} \end{array}$$

An active term is calculated from the difference of the left and right muscle activations $M_{i}^{l}$ and $M_{i}^{r}$ multiplied by a gain α. A stiffness term is calculated from the muscle activities, the tonic stiffness γ, a stiffness gain β and the joint angle ϕ_*i*_. A damping term is calculated from a damping constant δ and the joint angular velocity ${\dot{\phi }}_{i}$. Parameter values are given in Table 5.

**Table 5:** Muscle parameters.

**Name**	**Symbol**	**Value**
Muscle active gain (N·m)	α	0.4 (BTS, ST: 4^*^) (simulation) 0.5 (BTS, ST: 5^*^) (robot)
Muscle stiffness gain (N·m/rad)	β	1.2 (BTS, ST: 12^*^)
Muscle tonic stiffness (no unit)	γ	0.2
Muscle damping (N·m·s/rad)	δ	0.1

* *BTS, backward terrestrial stepping; ST, struggling*.

In simulations, a delay of 10 ms was introduced between the CPG outputs *x*_*i*_, *x*_*i*+25_ and the corresponding muscle activations $M_{i}^{l}$, $M_{i}^{r}$, respectively, as a minimum to account for the muscle activation dynamics. This delay was not necessary in robot experiments since the motor torque controller already introduces a larger delay of the order of 50 ms, which is consistent with the reported range (50 ms to 1 s) of the low-pass filter properties of muscle contraction (Partridge, 1965).

### Limb Joints

For the limbs, the oscillator phase θ_*i*_ is used directly as a representation of the desired position, with a piece-wise linear transfer function that modulates the swing and stance rotation speeds such as to obtain a duty factor of 77% (Ashley-Ross and Lauder, 1997; Ashley-Ross et al., 2009). For backward terrestrial stepping the direction of limb rotation was inverted by using −θ_*i*_ instead of θ_*i*_.

### Sensory Feedback

Proprioceptive feedback signals *s*_*i*_ are derived from the joint angles ϕ_*i*_ by simulating the activity of stretch receptors: $s_{i}=w^{ipsi}{s_{i}}^{ipsi}+w^{contra}{s_{i}}^{contra}$, with ${s_{i}}^{ipsi}$ and ${s_{i}}^{contra}$ the positive part of ϕ_*i*_ and −ϕ_*i*_, respectively, for the left side (−ϕ_*i*_ and ϕ_*i*_ for the right side), and *w*^*ipsi*^ and *w*^*contra*^ the feedback weights. Since the axial part of the CPG model has 25 segments (each containing 2 hemisegmental oscillators) and the robot only 8 active axial joints (Figure 1B), some mapping is necessary. The signal from each joint is sent to the 3 neighboring segments, while only the middle segment is used to drive the joint muscles (Figure 1B). This leaves segments 3 and 16 without feedback, which is reasonable since the amplitude of the body curvature is smallest at these positions in the animal (Karakasiliotis et al., 2013).

In some simulations, an additional term was added to the ${\dot{\theta }}_{i}$ equation for limb oscillators to represent excitatory proprioceptive feedback from the limbs, as used in a previous study (Harischandra et al., 2011). Here a simplified form was used:

$$\begin{array}{l} {\dot{\theta }}_{i}=2\pi \nu_{i}+\underset{j}{\sum }r_{j}w_{ij}\sin\left(\theta_{j}-\theta_{i}-\varphi_{ij}\right)\\ \;+w^{limb}\max\left(0,1-\frac{\left|\phi_{i}-\phi_{i}^{0}\right|}{\frac{\pi }{2}}\right) \end{array}$$

Here *w*^*limb*^ is the feedback weight, ϕ_*i*_ the joint angle of the robot rotational limb and $\phi_{i}^{0}$ the angle at the transition from stance to swing. The feedback is maximal at the end of the stance and decreases linearly on either side until it reaches zero. The rate of decrease is such that the feedback is non-zero for half of the leg rotation. The value is always positive or zero, so this feedback term can only have an accelerating effect.

### Mechanical Simulation

3D simulations of the robot were performed using the Webots 6 software (Cyberbotics, Switzerland), which is based on the Open Dynamics Engine (ODE, www.ode.org). The physics engine was extended with a hydrodynamics model that includes reactive and resistive forces (Porez et al., 2014). The passive tail fin was modeled as a chain of 10 small segments with passive stiffness. The physics was simulated with a time step of 0.5 ms. The robot controller used a time step of 1 ms, the minimum value supported by Webots. This was just too high for a stable simulation of the muscles, so the physics plugin was used to implement the muscle model and set the joint torques.

### Robot Hardware

The robot *Salamandra robotica II* (Crespi et al., 2013) is made of a head module (9.6 cm long), 8 active modules (9.6 cm long each) and a 24.6 cm long, passive, flexible caudal fin (Figure 1A). This allowed the robot to approximately reach the tail length/total body length ratio of the real animal (around 0.5–0.6, see Ryczko et al., 2015). Each module actuates an axial joint with motion restricted to the horizontal plane; the two girdle modules also include rotational joints for the limbs. The entire robot measures 111 cm and weighs 2.48 kg. The robot modules have LEDs on the covers which were used to track the robot's motion with two Basler A622F video cameras (15 frames/s) to cover the whole track length (6 m) with an accuracy of ± 1 cm. The Supplemental Movies of the robot were captured with another camera at 15 or 30 frames/s. Two adaptations were made to the robot to reproduce the different behaviors. During forward underwater stepping, the buoyancy was adjusted by adding 72 g of lead in the head. This corresponds to +2.9% of the total robot weight, or + 41.8% of the normal weight of the head module (172 g). During struggling, tape was added under the feet to increase slipping, mirroring the conditions of the animal experiments (Ryczko et al., 2015).

### Distributed Electronics and Control Software

The robot controller is distributed: each module reads the position and velocity of the local joint and computes the control loop for the corresponding part of the CPG and the joint's virtual muscles, with a time step of 10 ms. The numerical integration of CPG segments with floating-point operations required a modification to the hardware described by Crespi et al. (2013): the modules were upgraded to include an LPC2129 ARM7TDMI microcontroller running at 60 MHz, as already present in the head. Communications between modules are restricted to drive signals from the head and CPG couplings between adjacent modules, sent over the CAN bus running at 1 Mbps. The leg positions are set by PD controllers using the motor encoders. The axial torques are set by PI controllers using current sensing. The CPG state from each module was recorded by logging coupling and debug messages sent over the CAN bus. This logging was done on an external computer, by spying on the bus using long, thin wires attached to the caudal end of the robot. Two modifications were made to the distributed controller between the initial tests and the final version (see Results): (1) The numerical integration of the CPG was changed to estimate the phases θ_*j*_ and amplitudes *r*_*j*_ of coupling sources at the time of integration using a linear extrapolation of the values from the two latest CAN messages and their times of arrival (coupling terms are dropped entirely from the integration if the two previous CAN messages are older than 200 or 400 ms, respectively); (2) The phases and amplitudes were encoded in CAN messages as 16-bit half-floats rather than 32-bit floats, such that coupling signals from a module to a particular neighbor would fit in a single message.

### Selection of Parameter Values

The CPG parameters were hand-tuned to reproduce the distribution of intersegmental phase lags and cycle durations from *in vitro* recordings of isolated spinal cords (Ryczko et al., 2015). To emulate the motor behaviors displayed by the animal *in vivo*, the CPG model was subjected to higher excitatory drives which were tuned to reproduce typical electromyographic patterns for each of the five motor behaviors, in terms of cycle frequency and intersegmental phase lags (Ryczko et al., 2015). However, the cycle frequencies targeted with the model were set to half that of the *in vivo* recordings. This was chosen to reflect the scaling of locomotion frequency with body mass observed in animals (Bejan and Marden, 2006). For robot experiments, the target frequency for swimming was further lowered to 1.1 Hz due to the limits of operation of the robot (in particular torque limits). Other than the drive levels and sensory feedback, the only changes from *in vitro* to *in vivo* CPG conditions were in the average saturation thresholds which had to be increased to match the higher drives used *in vivo*.

The parameter space for the virtual muscles and proprioceptive feedback was explored systematically using the 3D mechanical simulation of the robot during swimming. An “average individual” was used by setting the standard deviations of the CPG excitabilities to zero, to increase reproducibility (this restriction was relaxed for robot experiments). We used uniform muscle parameter values for trunk joints, and progressively smaller values in the tail to emulate body taper: the values of α and β in modules 6, 7, and 8 were multiplied by a factor 0.7, 0.5, and 0.2, respectively. The same feedback parameter values were used for all joints, and the same feedback weights (with opposite signs) were used for ipsilateral and contralateral projections. Initial tests were made with a tonic muscle stiffness γ = 0: this parameter is mostly redundant with the stiffness gain β for a given (non-zero) amplitude of CPG oscillations. A uniform excitatory drive was used for all oscillators, which in absence of feedback results in high intersegmental phase lags inappropriate for swimming. The drive was set to 1.34, corresponding to a swimming frequency of 1.47 Hz (in absence of sensory feedback), which is close to our target of half the frequency observed in the animal (2.78 Hz and 3.12 Hz during EMG and kinematic recordings, respectively, Ryczko et al., 2015). We selected muscle and feedback parameter values that showed a significant increase in swimming speed and high stability of the CPG and kinematic patterns, while keeping the joint torques close to the robot's limit of 0.7 Nm (Supplementary Figures 1–3).

The five salamander behaviors were first reproduced in simulation without sensory feedback, by tuning the CPG drive levels and optimizing the limb-body phase bias for speed of locomotion. Additional simulations were done with varying strengths of axial proprioceptive feedback during forward terrestrial stepping and swimming. Further simulations were made with proprioceptive limb feedback during forward terrestrial stepping.

The limb-body phase bias was optimized again on the robot, due to the different zero-point reference and the backlash in the gears. The main simulation results were then reproduced on the robot with five different “individuals,” which were modeled by initializing the CPG parameters using different random seeds. The descending drives were adjusted for each “individual” and each motor behavior. Other parameters were sometimes adjusted between behaviors but always using the same values for all individuals (see Results). The movies shown in the Supplemental Materials were prepared using an average individual.

### Data Processing

The joint angles from simulations and robot experiments were calculated in Matlab by fitting the kinematic chain of the robot to the positions of the LEDs. The CPG and kinematic intersegmental phase lags were calculated in Matlab from the CPG output and joint angle oscillations using the same algorithm: The timing of each cycle was defined as the centroid of the positive part of each oscillation (Knüsel et al., 2013). These timings were used to calculate a median phase lag (over time) for each pair of consecutive segments (CPG lag) or consecutive joints (kinematic lag). The CPG intersegmental phase lag was calculated using the average of the median phase lags between segments 8 to 12. For simulations, the kinematic intersegmental phase lag was calculated using the average of the median phase lags between joints 3 to 5. For robot experiments, joint 5 was often an outlier due to the robot torque limits, so joints 2 to 4 were used instead.

### Statistics

Data are given as means ± standard deviation (SD) unless specified otherwise. Correlations between variables were evaluated in SigmaPlot 11.0 using the Pearson Product Moment Correlation test.

## Results

### The Isolated CPG Model Reproduces the Main Features of Recordings From Isolated Spinal Cords

**Figure 3:**
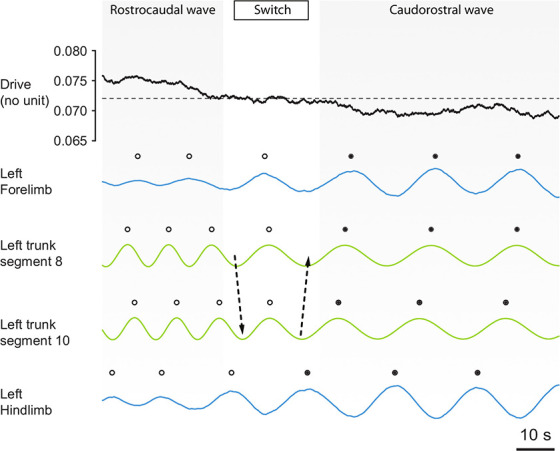
The CPG spontaneously switches between axial motor patterns as a function of a fluctuating background drive strength. The strength of the simulated pharmacological drive applied to the CPG is shown together with the outputs of the oscillators of the left forelimb, left trunk segments 8 and 10, and the left hindlimb. Before the switch, limb oscillators (blue lines) are saturated by the drive strength. Therefore, they show low amplitude oscillations and are entrained to the higher frequency of the trunk oscillators, and the motor pattern in the trunk segments (green) is a rostrocaudal wave (white dots). Then, a progressive decrease in drive strength occurs through random fluctuations, and this progressively de-saturates the limb oscillators. The de-saturation allows limb circuits to oscillate at higher amplitude, causing a switch (arrows) from a fast rostrocaudal wave to a slower caudorostral wave (black dots). After the switch, the de-saturated limbs show high amplitude oscillations and therefore entrain to their slower frequency the trunk oscillators, because effective connection strength in the model is proportional to the amplitude of oscillations from the sender (see Methods). The same switches have been observed in the isolated spinal cord [Figures 5A,B of Ryczko et al. (2015)].

We found that asymmetric intersegmental couplings, together with different excitabilities and saturation thresholds between forelimb, hindlimb and axial oscillators, enabled the spinal cord model to reproduce the trimodal distribution of phase lags observed *in vitro* during fictive locomotion (Ryczko et al., 2015). The values shown in Tables 2, 3 were found to produce a range of phase lags similar to the biological data, with peaks centered on 6.7 ± 1.3, 1.3 ± 1.3, and −6.1 ± 1.0% (Figures 2B,C). The positive correlation between phase lag and cycle duration (Ryczko et al., 2015) was also reproduced (Figure 2D). Furthermore, the small fluctuations in the excitatory drive over time allowed the model to reproduce the spontaneous switches between slow caudorostral waves and fast rostrocaudal waves of axial activity reported in the isolated spinal cord (Delvolvé et al., 1999; Ryczko et al., 2015). Figure 3 shows an example of the model producing such a switch.

### Improving Swimming With Proprioceptive Feedback Requires Specific Muscle Stiffness and Damping Properties

Using a uniform drive yielded an intersegmental phase lag of 6.6% in an average individual (leftmost peak of the distribution). This resulted in inefficient swimming, with too many nodes in the traveling wave (Supplementary Movie 1). We looked for muscle parameters that would allow proprioceptive feedback to improve swimming by decreasing the phase lag toward more physiological values. For the active gain α, a value of 0.4 proved optimal, as higher values (together with higher stiffness β or γ) would have given higher swimming speeds but would have required torques beyond our robot's limits. Systematic tests in the β, δ, *w*^*ipsi*^ space showed a single region where feedback increased the swimming speed thanks to a decrease of the phase lag, while keeping the CPG rhythm stable. This stable region corresponds to a stiffness gain β between 1.6 and 2.3, a damping δ between 0.05 and 0.15 and feedback weights *w*^*ipsi*^ = −*w*^*contra*^ between 17 and 22 (Supplementary Figures 1–3). Further tests in the β, γ, *w*^*ipsi*^ space showed that we could trade some fitness gain β for tonic stiffness γ. We settled on β = 1.2, γ = 0.2, which give qualitatively reasonable passive mechanical properties (Table 5).

### Simulation Results Transferred to the Robot Following Some Adaptations

The simulation results could be reproduced on the robot, with the following changes made based on qualitative judgements: the muscle active gain α had to be increased from 0.4 to 0.5 to obtain reasonable amplitudes of oscillation during swimming. Uniform muscle parameter values were used in all robot joints: the tapering of the active and stiffness gains was removed to obtain reasonable amplitudes of oscillations in the tail and good swimming speeds. Due to the limits of operation of the robot, the target frequency for swimming had to be lowered down to 1.1 Hz^2^. The feedback weights were reduced to 10. Sensory feedback from the neck joint was removed as it was destabilizing, leading to aperiodic rhythms. Finally, in simulation we found that a common limb-body phase bias gave near-optimal speed for all stepping behaviors. This was not the case with the robot: a specific limb-body phase bias was required for backward stepping (Table 2) to obtain the optimal speed for that behavior.

### Two Drive Signals Suffice to Reproduce the Five Motor Behaviors, but Backward Terrestrial Stepping and Struggling Require Stronger Muscle Contractions

**Figure 4:**
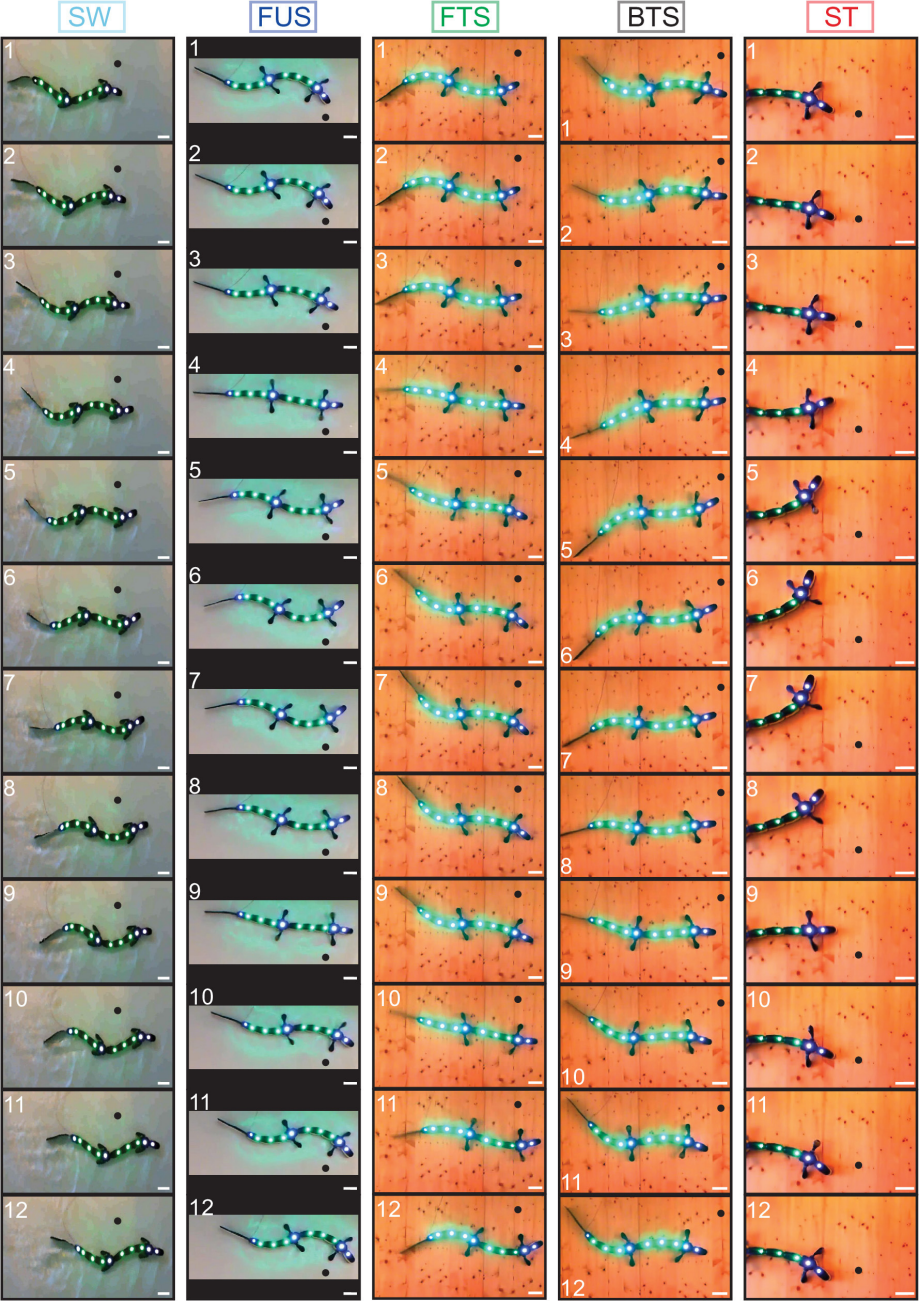
Frame sequences showing from top to bottom one complete stride for the five motor behaviors. Light-emitting diodes were tracked on each module for kinematic analysis. A black dot was added on each frame to illustrate robot progression. Inter-frame time intervals in ms are, respectively: 200 (swimming, SW); 133.3 (forward underwater stepping, FUS); 166.7 (forward terrestrial stepping, FTS); 400 (backward terrestrial stepping, BTS); 466.7 (struggling, ST). Scale bar (white), 10 cm. The different background colors are due to the different environments: water tank for SW and FUS, wooden board for the other behaviors. The behaviors are close to those observed in the real animal [Figure 1 of Ryczko et al. (2015)].

**Figure 5:**
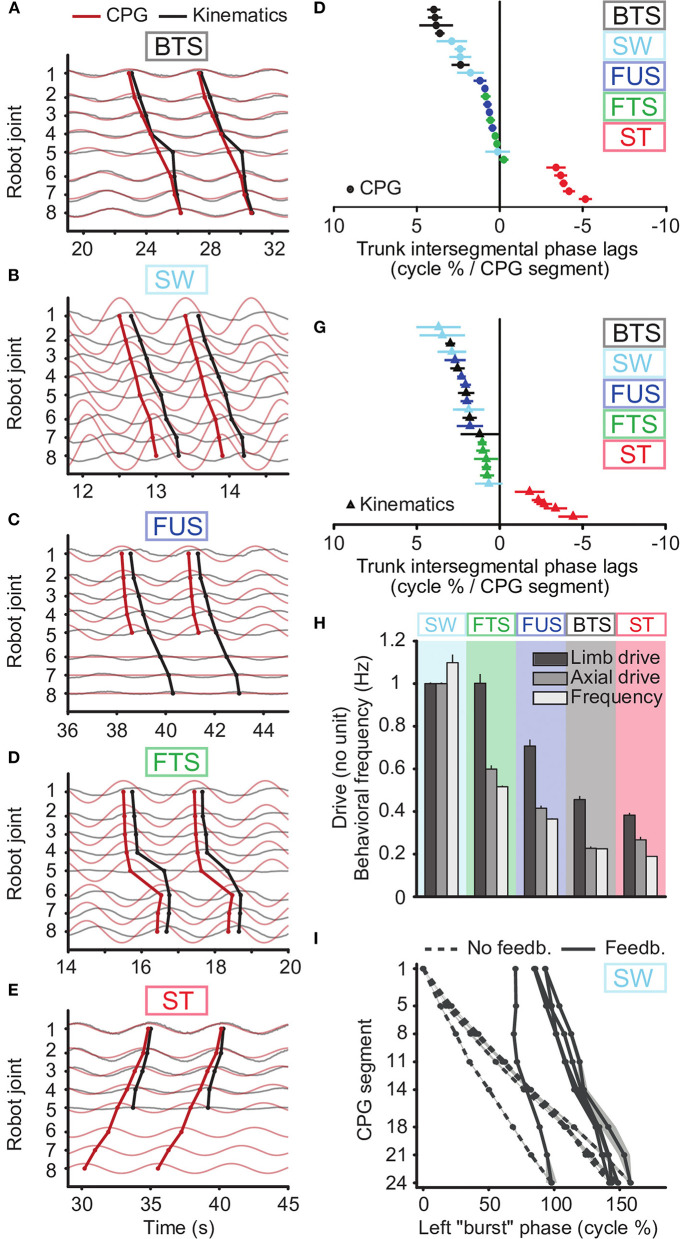
CPG output and joint kinematics during the five motor behaviors reproduced with the robot. Results obtained using a single drive level and proprioceptive feedback for swimming, two drive levels (to limb and axial oscillators) and no feedback for the other behaviors, with a third drive of zero for the tail during underwater stepping. **(A–E)** and **(F,G)** emulate the biological data illustrated in Figures 2, 3 of Ryczko et al. (2015), respectively. **(A–E)** Kinematic angular oscillations (thin black lines) and CPG outputs *x*_*i*_ (thin red lines) are shown for each joint, with circular markers indicating the centroid of the positive half of each cycle. Rostrocaudal (A-C, line descending to the right), standing (D, almost vertical line) and caudorostral (E, line descending leftward) kinematic waves (thick black lines) followed the CPG activity (thick red lines) with variable delays during the 5 motor behaviors: backward terrestrial stepping (BTS), forward swimming (SW), forward underwater stepping (FUS), forward terrestrial stepping (FTS) and struggling (ST). All figures are from the same simulated individual. **(F,G)** Intersegmental phase lag distributions for CPG waves **(F)** and for kinematic waves **(G)** in the robot trunk. Each marker represents a single recording. **(H)** Comparison of the tonic drive signals applied to the CPG and resulting oscillation frequencies (Hz) for each motor behavior. Data represent mean ± SD over the 5 simulated individuals. The overlap in limb drive values between SW and FTS is due to reduced drive and limb saturation thresholds during SW to accommodate for the robot's torque limitations (See Methods). Without these limitations, the limb and axial drives for SW would be 1.34 as in simulation. **(I)** Rostrocaudal axial waves generated during SW by the CPG without sensory feedback (dashed lines) and with sensory feedback (solid lines), with uniform drive *d*_*i*_ = 1, in the 5 simulated individuals. For each individual, the wave with feedback is horizontally positioned in the figure to connect to the wave without feedback. The experiments with sensory feedback correspond to the SW data in **(F–H)**.

We found that tonic drives with only two different values applied to different parts of the CPG were sufficient to reproduce qualitatively the five motor behaviors with the robot, as shown by movies (Supplementary Movies 2–6), frame sequences (Figure 4) and robot kinematics (Figure 5, Supplementary Figure 4B). In particular, the differences in CPG and kinematic intersegmental phase lags between the five behaviors were reproduced (Figures 5F,G), as well as the differences in cycle durations (Figure 5H). Figures 5A–E show the CPG outputs and joint oscillations for a single individual. The rostrocaudally increasing lag between CPG and kinematic waves is reproduced (increasing gap between the thick red and thick black lines).

Swimming could be obtained by sending a strong drive (i.e., saturating limb oscillators, Hypothesis 3) to the whole CPG, with a slightly lower drive to the most rostral oscillators (segments 1-3 in Figure 2) to adjust the phase lag as proposed in numerical simulation of the lamprey locomotor CPG (Kozlov et al., 2009). The other behaviors were obtained by adjusting the drive to the limb oscillators independently from the drive to the axial oscillators (Figure 5H, Supplementary Movies 3–6). Higher axial phase lags required a greater relative difference between the two drives, and higher frequencies required higher values of both drives.

While two drives were sufficient to generate the CPG activity patterns for all behaviors, we found that stronger forces from the virtual muscles were required to reproduce the kinematics of backward terrestrial stepping and struggling. To avoid introducing additional parameters, this was implemented by increasing the muscle gains α and β. A 10-fold increase was found appropriate to avoid large deviations between the CPG activity and the kinematics (Table 5, Figures 5A,E).

### Proprioceptive Feedback Can Regulate the Phase Lag During Swimming and Forward Terrestrial Stepping, and Reduces Variability

With axial proprioceptive feedback, swimming could be reproduced with a single drive to the whole CPG (Figures 5B,I, Supplementary Movie 8). In absence of feedback, the axial network of the five robot “individuals” produces intersegmental phase lags of 6.1 ± 1.4% (Supplementary Figure 4A, Supplementary Movie 1). With feedback, this could be reduced to 1.9 ± 1.1%, which matches the values observed in the animal (1.89 ± 0.25%, Ryczko et al., 2015). Feedback also reduced the variability between individuals: Without feedback, the individual corresponding to the leftmost curve in Figure 5I (dashed lines) was an outlier. Using feedback with identical weights in all individuals (solid lines), the outlier showed phase lags similar to the other individuals.

**Figure 6:**
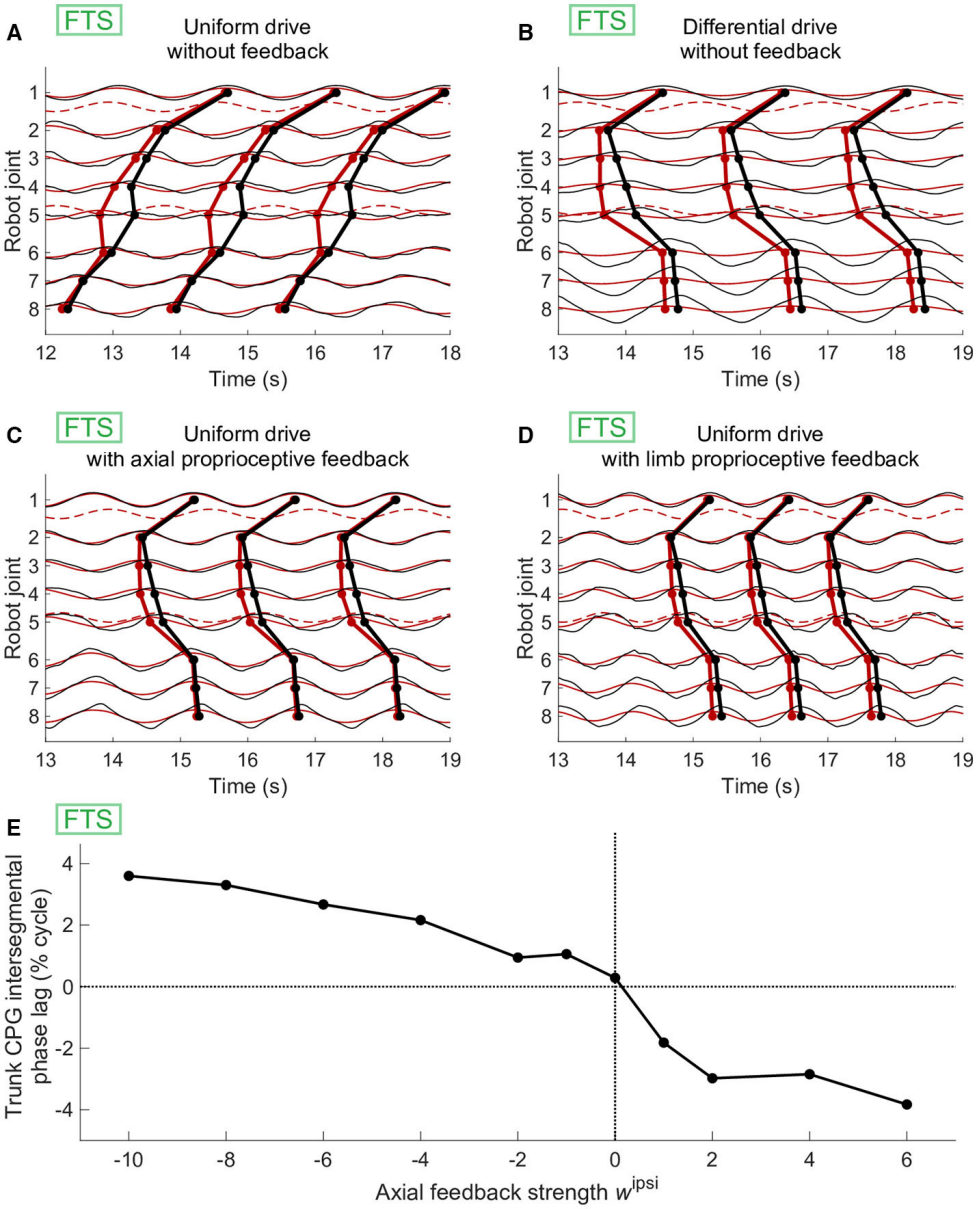
Effect of axial or limb proprioceptive feedback on CPG activity and kinematics during forward terrestrial stepping (FTS). **(A–D)** Joint kinematics (black) and CPG activity (red, dashed for limb oscillators) in simulation. Circular markers indicate the centroid of the positive half of each cycle. The limb-body phase bias φ_*ij*_ was adapted in each case for optimal speed of locomotion. **(A)** With uniform drive (*d*_*i*_ = 0.98) and no feedback, the CPG with active (non-saturated) limb oscillators produces caudorostral waves of activity. **(B)** Standing waves of CPG activity can be obtained by using a different drive of 0.63 for the limb oscillators. **(C)** Standing CPG waves can also be obtained using uniform drives and axial proprioceptive feedback, with *w*^*ipsi*^ = −*w*^*contra*^ = −0.65. **(D)** Standing waves of CPG activity could also be obtained with uniform drives using proprioceptive limb feedback, with *w*^*limb*^= 3.7. **(E)** Effect of the axial feedback weight on CPG axial intersegmental phase lag in robot experiments. Before introducing feedback, differential drives to the limb (*d*_*i*_ = 0.98) and axial (*d*_*i*_ = 0.61) oscillators were used to increase the phase lag toward zero. Positive ipsilateral feedback weights (as those used during swimming with sensory feedback, Figure 5I) decreased the phase lag, whereas negative ipsilateral weights increased it.

Axial proprioceptive feedback could also replace differential drives (i.e., different drive values for different parts of the CPG) as a means of obtaining axial phase lags close to zero in simulations of forward terrestrial stepping. However, this required feedback weights of opposite signs compared to swimming. Using uniform drives and no feedback, the CPG generated negative phase lags (Figure 6A). With uniform drives, positive values of *w*^*ipsi*^ (as used in swimming) had a destabilizing effect on the CPG, with oscillators failing to lock in frequency. With differential drives as in Figure 6B, we found that positive values of *w*^*ipsi*^ were counterproductive, decreasing again the CPG phase lag to negative values. However, negative values of *w*^*ipsi*^ produced higher phase lags as desired and could be used to reproduce forward terrestrial stepping with uniform drives (Figure 6C). This effect of an increasing intersegmental phase lag with decreasing feedback weights during forward terrestrial stepping was reproduced in robot experiments (Figure 6E).

Instead of axial feedback, the differential drive could also be replaced with limb proprioceptive signals fed back to the limb oscillators. This was only tested in simulation (Figure 6D). The effect of feedback here was again to increase the negative CPG phase lags toward slightly positive values, close to zero.

### A Passive Tail Decreases the Drag During Underwater Stepping

**Figure 7:**
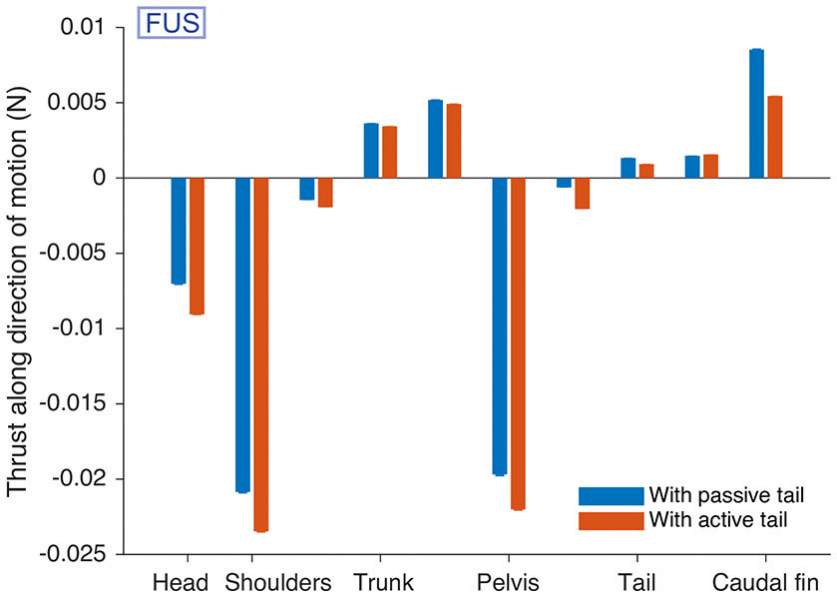
Passive tail CPG segments reduce drag during forward underwater stepping (FUS) in simulation. The hydrodynamic forces acting on different parts of the robot modules along the direction of motion are given. The caudal fin provides more thrust when the tail is passive. Forces on each module were measured for an “average individual” during 15 s after a warm-up period of 15 s, and averaged over the 30,000 time steps. The procedure was repeated 100 times with different starting conditions. The bars show the means over the 100 repetitions. Error bars for the standard error are shown, but barely visible (all standard errors are < 0.00013).

During forward underwater stepping, the locomotor performance could be improved by using “passive” tail segments (Supplementary Movie 7), similar to the animal which shows passive tail undulations during this locomotor behavior (see Cabelguen et al., 2014). This was implemented using a third drive level of zero to the tail segments. The tail CPG was then inactive, but the robot modules continued to generate torques corresponding to the passive parts of the muscle model (the terms that remain when $M_{i}^{l}$ and $M_{i}^{r}$ are 0). Measurements of the hydrodynamic forces in the 3D mechanical simulation suggest that for this particular gait, the undulations of passive tail segments allow the caudal fin to generate more thrust than in the case of active tail segments (Figure 7). The drag at the head and girdles is also reduced in the passive case. The net drag on the body axis is thus reduced from −0.0422 ± 0.0003 N to −0.0295 ± 0.0004 N (standard errors).

### Coupling Delays Introduce Systematic Phase Biases in the Distributed Robot Controller

**Figure 8:**
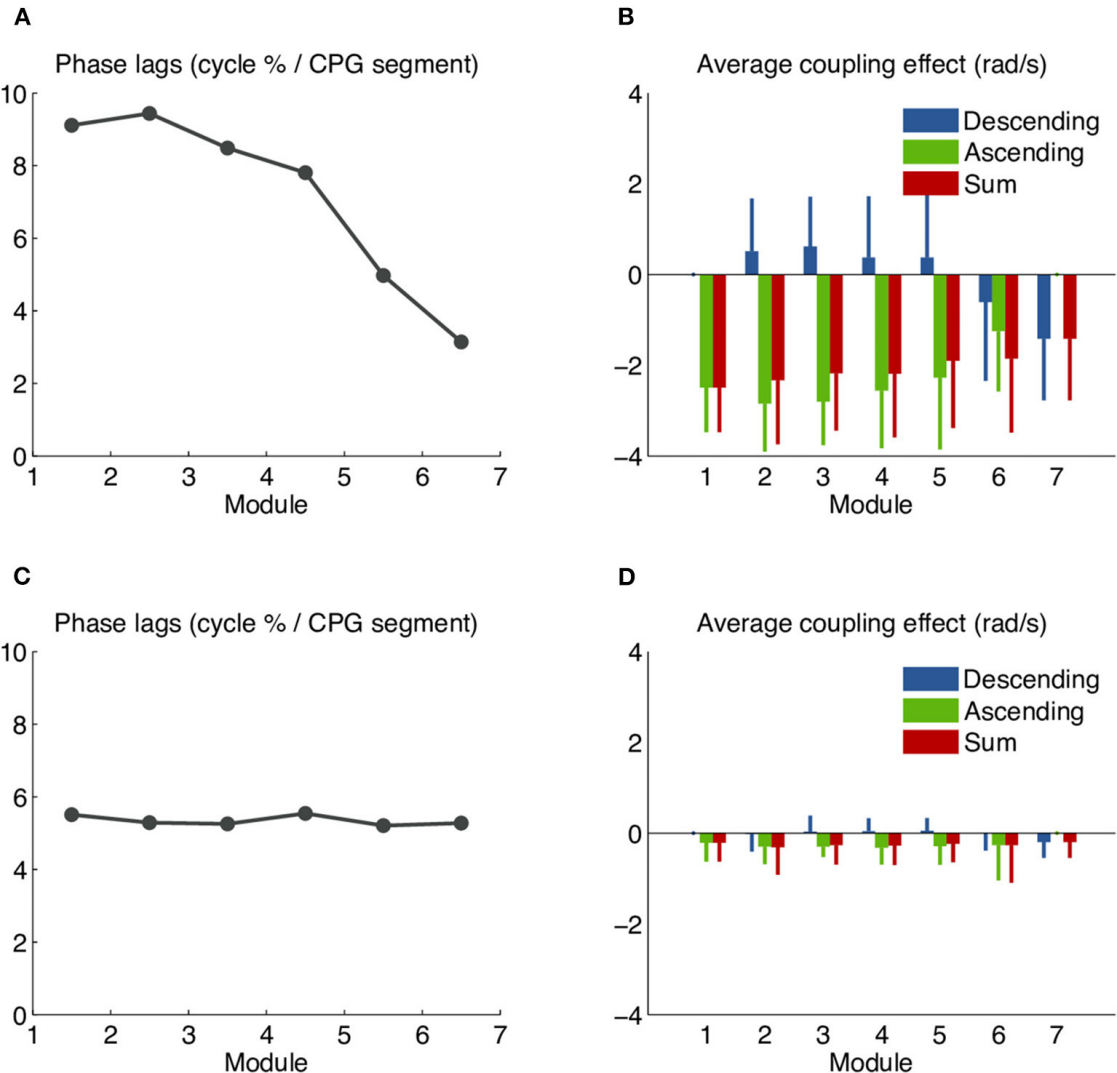
Systematic phase biases due to the slow-down effect of coupling delays in the robot's distributed controller. Undesired phase lags in the distributed controller were analyzed using a chain of 7 simple (non-girdle) robot modules and a CPG model with symmetrical ascending and descending coupling weights and a uniform intersegmental phase bias of 5%. **(A)** CPG intersegmental phase lags calculated from the middle segment of each module, as percentage of the cycle duration. The observed values are higher than the target of 5% in the rostral modules and lower than the target in the caudal ones. **(B)** Average effect of the ascending and descending couplings on the left oscillator of the middle segment in each module. Values shown correspond to the coupling terms *r*_*j*_*w*_*ij*_sin(θ_*j*_ − θ_*i*_ − φ_*ij*_), averaged over the whole recording. The negative red bars show that the net effect of both coupling types is to slow down the oscillation. The effect is stronger in more rostral modules. **(C)** Phase lags observed after the implementation of coupling message extrapolation. The observed values are almost uniform and close to the target of 5%. **(D)** With coupling message extrapolation, the slow-down effect has almost vanished.

Initial tests on the robot with the distributed controller gave non-uniform phase lags along the body, unlike what was seen in simulation. We investigated the issue using a chain of 7 simple modules (no girdles) and a CPG model with symmetrical ascending and descending coupling weights and phase biases of 5%. We found increased phase lags between the first modules and decreased phase lags between the more caudal ones (Figure 8A). An analysis of the coupling terms used in the numerical integration of the CPG showed that rostral modules were significantly slowed down by caudal ones (Figure 8B). This suggested that the θ_*j*_ values (the phases of the couplings' source oscillators) used in the numerical integration of the target oscillators were out of date. We modified the numerical integration to estimate the state of the source oscillator at the time of integration using a linear extrapolation of the two previous coupling messages and their times of arrival (see Methods). This considerably reduced the slow-down effect and yielded almost uniform phase lags (Figures 8C,D). Further improvements (not shown) were obtained by encoding the coupling phases and amplitudes as 16-bit half-floats. This halved the number of messages sent over the CAN bus and helped decrease the rate of transmission errors.

### The Distributed Controller Allows for Autonomously Moving Robot Parts

**Figure 9:**
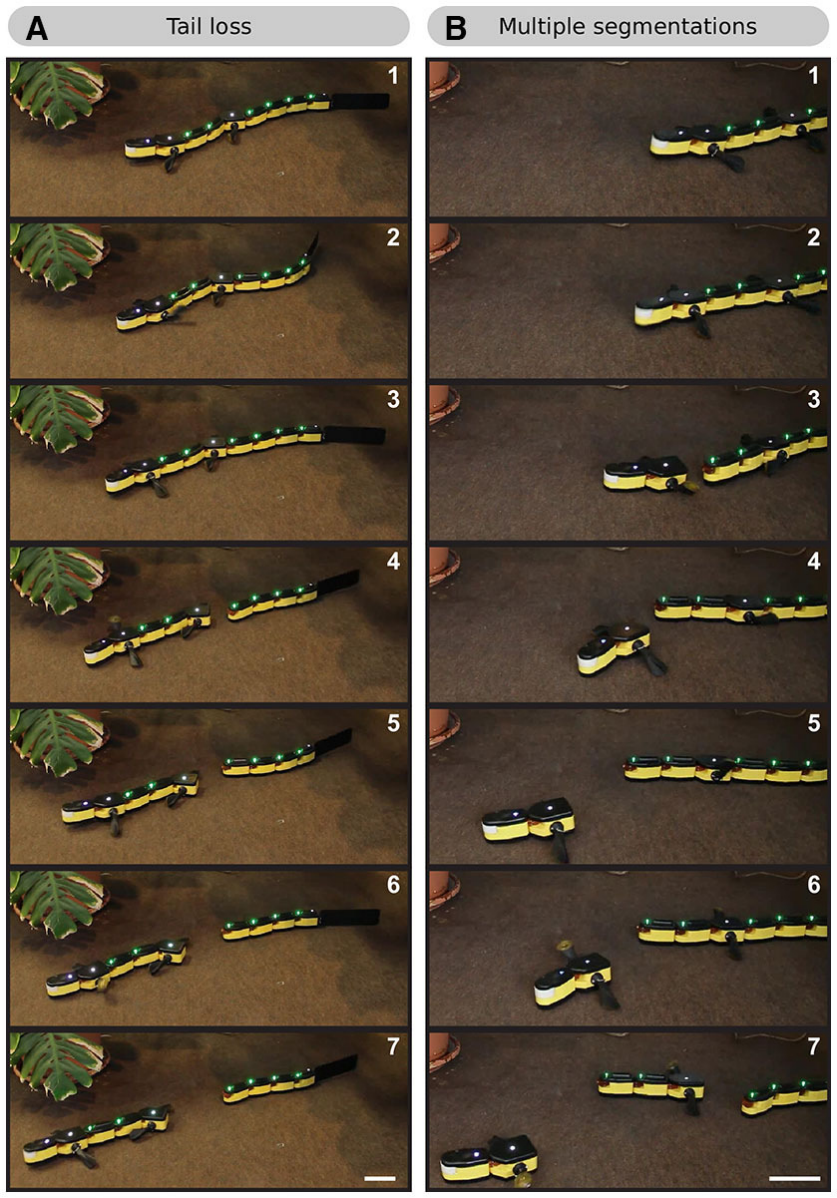
Frame sequences showing the behavior of the robot when split into several parts. **(A)** After losing its tail, the robot keeps moving (although with a malfunction in the pelvic girdle module). The tail modules continue to oscillate. The tail CPG maintains the coordination in the form of a rostrocaudal traveling wave. **(B)** The robot splits behind both girdles. The three robot parts keep moving independently.

We found that the distributed implementations of the CPG and muscle models have the side effect of making the robot modular at runtime. We conducted forward terrestrial stepping experiments with screws between some modules removed, causing the robot to break into parts (no other changes were made to the hardware or software). Each part kept functioning, still coordinated by its own section of the CPG model. This is demonstrated in Supplementary Movie 9, Figure 9.

## Discussion

### A Modulable CPG Architecture

Our results suggest that the answer to question 1 is yes: a modulable CPG provides a robust framework for generating multiple motor patterns, such that different motor behaviors do not necessarily require dedicated CPGs. This concept was proposed by Grillner (1981) as the “unit burst generator” theory, which states that independent rhythmogenic circuits can be flexibly coupled from one behavior to another. Such circuits have been identified in many animals. In the salamander, specific spinal hemisegments have been shown to control muscles of the trunk (Ryczko et al., 2010), tail (Charrier and Cabelguen, 2013) and limbs (Cheng et al., 1998; Lavrov and Cheng, 2004; Ijspeert et al., 2007). Other examples include the spinal hemisegments in the lamprey (Cangiano and Grillner, 2003, 2005; Cangiano et al., 2012), the flexor and extensor networks in the left and right side of the mouse spinal cord (Hägglund et al., 2013), the crayfish swimmeret system (Mulloney and Smarandache-Wellmann, 2012) and the networks controlling individual leg joints in the stick insect (Büschges et al., 1995). A modeling study of insect locomotion suggests that the recruitment of a single neural structure for various behaviors also applies to situations where locomotion is largely driven by sensory feedback (Schilling and Cruse, 2020).

### Oscillator Couplings

Biological data indicate that in salamanders, limb activity can occur together with traveling waves *in vivo* and *in vitro* (Ryczko et al., 2015). In our previous robotic study (Ijspeert et al., 2007), limbs projected to all axial oscillators (forelimb oscillators to trunk oscillators, and hindlimb oscillators to tail oscillators). Two axial outputs were therefore possible: either a standing wave when limb oscillators were active (during stepping), or a rostrocaudal wave when limbs were saturated (during swimming). Here we made the limb oscillators project only to neighboring axial oscillators (Hypothesis 1), which gives more flexibility for the coordination of axial oscillators when limbs are rhythmically active (Ijspeert et al., 2005; Knüsel et al., 2013). In the animal, a unidirectional connection from excitatory interneurons generating the limb rhythm to those generating the axial rhythm would be sufficient to impose the slow limb oscillations to the axial segment, according to a detailed model of salamander spinal networks based on Hodgkin-Huxley neurons (Bicanski et al., 2013).

In our model, the asymmetry between ascending and descending coupling weights *w*_*ij*_ is required to reproduce a wide diversity of axial phase lags with consistent values along the axis as observed *in vitro* and *in vivo* (Ryczko et al., 2015). Symmetric weights as used in Ijspeert et al. (2007) produce non-uniform phase lags along the axis when the oscillators have different intrinsic frequencies. Future studies should determine whether such a coupling is present in salamanders and how it is implemented. Possibilities include an asymmetry at the neuroanatomical level (dominance of descending projections, see Buchanan et al., 1989; Buchanan, 2001 in lamprey) or in electrophysiological terms (stronger synaptic strengths toward caudal segments, see Smarandache et al., 2009 in crayfish; more spikes per locomotor cycle in neurons projecting caudally, see Mulloney et al., 2006 in crayfish).

### Oscillator Frequencies and Saturation

For our CPG model to be able to generate the three types of axial waves recorded *in vitro* and *in vivo* in salamanders, and the positive correlation between cycle duration and phase lag (Ryczko et al., 2015), we had to modify the intrinsic frequency of limb networks compared to our previous study (Ijspeert et al., 2007). Forelimb and hindlimb oscillators still have an intrinsic frequency slower than axial oscillators, but here forelimb oscillators are faster than hindlimb ones (Hypothesis 4). Data in mammals suggest that forelimbs deprived of normal interactions with the hindlimb networks tend to accelerate *in vivo*. Indeed, in adult cats where the spinal cord is partially lesioned, forelimb and hindlimb rhythms often dissociate, and forelimbs adopt a faster rhythm, yielding a 2:1 forelimb-hindlimb coupling (for review, see Frigon, 2017). At the cellular level, modification of a single conductance controlling burst termination should be sufficient to make limb segments generate slower oscillations, as suggested by a detailed Hodgkin-Huxley model of a salamander spinal segment (Bicanski et al., 2013).

A hypothesis that we kept from our previous work (Ijspeert et al., 2007) is that with a strong descending drive, limb networks “saturate” whereas axial oscillators do not (Hypothesis 3). Future studies should examine whether and how such a function is implemented in the animal. It could be a differential recruitment of specialized interneuron populations as a function of drive strength, as documented as a function of speed in zebrafish (McLean et al., 2007, 2008; Gabriel et al., 2011; Ampatzis et al., 2014, for review see Berg et al., 2018) and mice (Talpalar et al., 2013, for review see Kiehn, 2016). It could also involve a shift in the active set of reticulospinal neurons as a function of speed/gait. Some reticular neurons increase their firing specifically during swimming in salamanders (Lowry et al., 1996). Different reticulospinal neurons are activated as a function of speed in zebrafish (Kinkhabwala et al., 2011).

### Regulation Through Descending Drives

In answer to question 2, our results (Figure 5, Supplementary Figure 4B, Supplementary Movies 2–6) suggest that independent drive levels to a few parts of a CPG network (here two, or three to reproduce passive tail undulations) are sufficient to emulate a diversity of motor behaviors. In the model, the regulation of CPG activity by descending drives can be understood intuitively. The drive signals control the intrinsic (uncoupled) frequencies of the oscillators. Because rostrocaudal couplings are stronger than caudorostral couplings, a segment will entrain a slower or faster caudal neighbor, and the resulting common frequency will be close to the frequency of the rostral segment. However, the faster segment will lead the slower one with a delay that increases with the difference in uncoupled frequencies (this delay being in addition to the coupling's natural phase bias). This effect will propagate down the chain of segments, such that the resulting frequency and phase lag of the whole chain can be controlled by adjusting two values: the uncoupled frequency of the first segment, and that of the other segments.

This mechanism of regulation is close to the “trailing oscillator hypothesis,” which states that the oscillator of higher excitability becomes the leader of the chain. This hypothesis is based on lamprey experimental data showing that increasing the excitability in caudal segments causes a switch from a rostrocaudal to a caudorostral wave in isolated spinal cords (Matsushima and Grillner, 1990, 1992). However, this lamprey model assumed symmetrical rostrocaudal and caudorostral couplings, while we found that the coupling asymmetry is important to maintain a uniform phase lag along the chain of oscillators. A later lamprey modeling study with a detailed neural network of Hodgkin–Huxley neurons showed that dominant descending couplings allow for flexible control of forward and backward swimming with constant phase lag along the spinal cord at different speeds: the frequency and intersegmental phase lag can be controlled by adjusting the excitatory drive of the first segments compared to the remaining ones (Kozlov et al., 2009).

In our salamander model, the differential excitation of the first segments can be realized through the strong connections from the forelimb oscillators (when they are active). The regulation of the axial CPG pattern is then achieved by adjusting the excitation of the limb oscillators compared to the axis, instead of the first axial segments compared to the others as in the swimming case. This mechanism of regulation has been investigated with abstract oscillators and validated with a more detailed integrate-and-fire model (Knüsel et al., 2013).

The coordination of limb muscles was beyond the scope of this study: the limbs of our robot have a single rotational degree of freedom, and the direction of rotation was artificially inverted for backward stepping. We expect that more drives would be required in a model with more realistic limbs. Turning was also not investigated here but can in principle be obtained during swimming and stepping using different drives for axial oscillators on the left and right sides (Ijspeert et al., 2007).

### The Regulation Mechanism in the Isolated CPG

The mechanism of regulation described above, together with the differences in excitability and saturation thresholds between forelimb, hindlimb and axial oscillators (Table 3), enable the isolated CPG model to reproduce the trimodal distribution of phase lags observed *in vitro*: In the model, hindlimb oscillators are intrinsically slower than forelimb oscillators. Given the random nature of the saturation thresholds, forelimbs or hindlimbs can selectively saturate due to slightly different threshold values. When all oscillators are active, the hindlimb oscillators slow down the forelimbs, and the strong local connections from limb to axial oscillators slow down the girdle segments, leading to a highly negative phase lag in the trunk and tail axial networks. This corresponds to the rightmost peak of the distribution (i.e., negative lags, Figures 2B–D). When the hindlimb oscillators saturate, the forelimb oscillators accelerate a bit but continue to slow down the first segments, yielding the phase lags that make up the middle peak of the distribution (i.e., near zero lags, Figures 2B–D). When all limb oscillators saturate, the axial network is no longer influenced by limb network activity and generates the higher, positive phase lags found in the leftmost peak (i.e., positive lags, Figures 2B–D).

This mechanism also explains the spontaneous switches between slow caudorostral waves and fast rostrocaudal waves of axial activity: In the isolated CPG model, the transitions between the active and saturated states are triggered by small fluctuations in the excitatory drive (Figure 3), which represents tonic pharmacological excitation as in Ryczko et al. (2010, 2015) or Delvolvé et al. (1999). The progressive saturation of the limb oscillators causes their oscillation amplitude to diminish as the cycle frequency increases. The model thus suggests that limb burst amplitude *in vitro* should be higher during slow caudorostral wave of activity than during a rostrocaudal wave (Figure 3).

### Regulation Through Proprioceptive Feedback

Recordings from isolated spinal cords show much more variability among salamander individuals than EMG recordings of intact animals (Ryczko et al., 2015). In response to question 3, our results from robot experiments with five swimming “individuals” suggest that local sensory feedback could explain this reduction of variability from the *in vitro* to the *in vivo* condition (Figure 5I): sensory feedback made it unnecessary to tune the drive levels in each individual (compare the standard deviation of the drives in Figure 5H for swimming vs. the other behaviors). Results from robot experiments and simulations also suggest that local sensory feedback can replace differential drive as a modulator of the CPG activity to produce forward terrestrial stepping (Figure 6) and swimming (Figures 5B,I, Supplementary Figures 4A,C, Supplementary Movies 1, 8), which answers the other part of question 3.

The regulation of our CPG model by proprioceptive feedback can be explained with the same mechanism as regulation by different drive signals. Sensory feedback has been previously reported to increase the locomotion cycle frequency through an excitatory effect on the lamprey CPG activity (e.g., Kiemel and Cohen, 2001). In our model, the addition of proprioceptive feedback in the axis increases the uncoupled frequency of the segments in the axial network. If the first segment receives no feedback, as is the case in robot experiments, its uncoupled frequency is comparatively reduced. This leads as expected to a decrease in phase lags during swimming (Figure 5I).

Interestingly, simulations showed that feedback can also regulate swimming when neck feedback is included (Supplementary Figures 1–3, 4C). This suggests that feedback has a weaker accelerating effect in the first segment than in the others, even though the feedback amplitude is comparable (see Supplementary Figure 4C). This can be explained by looking at the model equations: axial feedback adds the term $-\frac{s_{i}}{r_{i}}\sin\theta_{i}$ to the instantaneous frequency ${\dot{\theta }}_{i}$ of the oscillator. The average value of this term is highly dependent on the phase relationship between θ_*i*_ and the phase of the feedback signal *s*_*i*_. In particular, if the kinematics follow closely the CPG output, and if we approximate *s*_*i*_ with a sine wave, then *s*_*i*_ will be proportional to cosθ_*i*_. Assuming a constant amplitude *r*_*i*_, the effect of feedback on ${\dot{\theta }}_{i}$ can be written *k*cosθ_*i*_sinθ_*i*_, which averages to zero over a 2π interval for θ_*i*_. We conclude that if θ_*i*_ increases approximately linearly with time, the effect of feedback on the frequency will approach zero when the CPG-mechanical phase lag approaches zero. And this lag (the distance between the red and black dots) is indeed very small for the neck joint in Supplementary Figure 4C.

We can also explain the need for reversed axial feedback weights during forward terrestrial stepping: Excitatory axial feedback (as in swimming) accelerates the mid-trunk oscillators, which tends to decrease the intersegmental phase lag. This is counter-productive since the unregulated intersegmental phase lag is already too low (Figure 6A). With inverted weights, axial feedback slows down the mid-trunk and increases the phase lag as desired. The inclusion of neck feedback has little importance in this case: when the limbs are active, the activity of segment 3 is largely determined by the strong connections from the forelimb oscillators, irrespective of the activity in the first and second segments. This also means that we would expect similar results with a model that includes head stabilization as observed in the animal during forward terrestrial stepping (Ryczko et al., 2015). Our results with inverted weights are reminiscent of the reversal of the effects of sense organs that signal forces on a leg when switching from forward to backward stepping in the stick insect (Akay et al., 2007, for review see Mantziaris et al., 2020). The mechanism underlying such a switch in sensory encoding could involve an interplay between the descending drive to the CPG and sensory feedback. In line with this possibility, brainstem stimulation changes how lamprey motoneurons respond to rhythmic movements imposed to the spinal cord (Hsu et al., 2013).

The regulation mechanism also explains the effect of limb feedback: the excitatory signal increases the frequency of the limb oscillators. These in turn increase the frequency of the first segments, and thus the intersegmental phase lag. Such limb feedback has been proposed in a simulation study as a way of facilitating the transition from walking to trotting in the salamander (Harischandra et al., 2011).

The cells underlying proprioceptive axial feedback remain to be identified in salamanders (see section Motor control in salamanders). The limb sensory feedback introduced in simulation could be provided by cutaneous receptors during foot contact since mechano-sensitive Merkel cells are present on the skin of salamanders (Scott et al., 1981, Diamond et al., 1986), and/or stretch receptors of limb muscles that are sensitive to joint angle, since fibers behaving as muscle spindles have been identified in salamanders (Bone et al., 1976). In mammals, it is well-established that limb feedback plays a key role in establishing the locomotor patterns (e.g., Musienko et al., 2012; Akay et al., 2014; Takeoka et al., 2014; for review see Frigon, 2017).

### Muscles and Passive Biomechanical Properties

We found that higher muscle torques were required to emulate struggling and backward stepping (Table 5). Behavior-dependent changes in limb electromyographic activity have been reported in salamanders when comparing forward and backward terrestrial stepping. The electromyographic bursts increase during backward stepping in the extensor iliotibialis pars posterior (the homolog of the rectus femoris in mammals, which elevates the femur and extends the knee), mostly during the swing phase, whereas the bursts decrease in the other limb muscles (Ashley-Ross and Lauder, 1997). Future studies should determine whether an increase in electromyographic activity occurs in axial muscles during backward terrestrial stepping. A differential ratio of activation of epaxial vs. hypaxial muscles in the animal could also occur, as observed when comparing forward underwater stepping and forward terrestrial stepping in salamanders (Deban and Schilling, 2009). The same comparative electromyographic measurements should be done for struggling in salamanders. Caudorostral waves of axial activity are also used during struggling in Xenopus and during backward swimming in eels and lampreys. Lateral body undulations are much larger during struggling and backward swimming than during forward swimming in Xenopus (Kahn and Roberts, 1982), in eel (D'Aout and Aerts, 1999) and in lamprey despite a similar duty cycle of the electromyographic burst (Islam et al., 2006), suggesting that an increase in muscle strength occurs during caudorostral waves.

Passive tail segments reduced the drag during forward underwater stepping in simulation (Figure 7). In line with this, tail muscles show weak or no activation despite large tail undulations during forward underwater stepping in salamanders, suggesting that the body generates thrust by transmitting trunk movements passively to the tail (Cabelguen et al., 2014). At the low frequencies of underwater stepping, the passive biomechanical properties of the tail could be sufficient to propagate the body undulation, while higher frequencies might require a higher stiffness and thus active muscles (Blight, 1976, 1977) as observed in the salamander during swimming (Delvolvé et al., 1997). In salamanders, whether tail deactivation during forward underwater stepping is due to reduced activity of e.g., some reticulospinal neurons remains to be determined. Biological observations and robotic experiments suggest that salamanders can use their tail as a “fifth limb” to provide thrust in slippery conditions (Karakasiliotis and Ijspeert, 2009). This suggests the existence of reticulospinal neurons that can both decrease and increase the activity of the tail independently from the trunk.

### Robotic Platform and Distributed Control

Some of the adaptations required to reproduce the simulation results on the robot can be explained by mechanical differences: The need for stronger muscle forces (no tapering) in the robot's tail might be due to the passive fin exerting more resistance than in the simulation. The different optimal limb-body phase bias for backward stepping could be due to the backlash in the leg gears.

Our initial implementation of the controller was centralized in the head module. This required retrieving the position and velocity of all joints and sending back the torque setpoints at each time step over the CAN bus. These are slow operations since the module has to forward the requests over a local I^2^C bus. The resulting control loop was too slow, making the muscle model unstable. The distributed controller solved this problem by keeping the communication of joint positions, velocities and torque setpoints local to each module. This solution shows interesting similarities with the vertebrate nervous system, which distributes the processing of sensory signals and the generation of locomotor patterns along the spinal cord, close to the target muscles.

Watanabe et al. (2009) have shown that a distributed controller with proprioceptive feedback can have interesting fault-tolerance properties, such as robustness to lesions in the communication pathways. It would be interesting to experiment with such lesions in our CPG model. The distributed controller would probably accommodate such experiments: the Supplementary Movie 9 shows that the different sections of the CPG continue to function after the robot has been split in several parts. This is an interesting feature that few robots have. It is made possible by the distributed computation of the CPG and muscle model, the multi-master nature of the CAN bus and the nearest-neighbor couplings of the CPG model.

The distributed controller also introduces a difficulty in the form of coupling delays, which can be hard to predict when many modules share the same communication bus. As illustrated in Figure 8A, these delays can have a significant impact on the coordination between modules: the phase lags between rostral modules are markedly increased, while those between caudal modules are decreased. The asymmetry is probably related to the priority of messages on the CAN bus: the last modules have higher CAN identifiers so lower priorities when several modules attempt to talk at the same time. This means that ascending couplings will be on average more delayed than descending couplings, inducing larger lags in the rostral modules (Figure 8B). The problem was mostly solved by extrapolating in the receiver module the state of the oscillators at the origin of the couplings (see Results). Extrapolating these states is easily done in our CPG model, where the state variables are the phase and amplitude of the oscillators: during steady state locomotion, these variables, respectively, grow at an almost constant rate or stay almost constant. It would be more difficult to extrapolate the state in a model without explicit phase variables. In conclusion, the CAN bus, being shared by all modules, limits the benefits of the distributed controller. A future revision of the robot should include direct communication between adjacent modules, in addition to the shared bus, to fully realize the benefits of distributed control.

## Conclusion

Following the analogy proposed by Loeb (2001), our study suggests that the spinal cord is as a puppet on strings, and that a complex motor repertoire can be generated by pulling a limited set of “sensory” or “descending” strings, which in turn take advantage of a flexible spinal motor circuit.

## Data Availability Statement

The raw data supporting the conclusions of this article will be made available by the authors, without undue reservation.

## Author Contributions

DR, JK, AI, and J-MC designed the study. JK and AC performed numerical modeling, simulations, and robotic experiments. JK, J-MC, AI, and DR analyzed data. JK, AI, and DR wrote the paper. All authors contributed to the article and approved the submitted version.

## Conflict of Interest

The authors declare that the research was conducted in the absence of any commercial or financial relationships that could be construed as a potential conflict of interest. The reviewer CL declared a past co-authorship with one of the authors AI to the handling editor.

## Footnotes

^1^When the excitatory inputs to the spinal cord increase beyond a certain level, CPG units saturate: they cease to produce rhythmic output. In the model, this is implemented as a decrease of oscillation amplitude toward zero (see the left part of Figure 3 for an illustration).

^2^This meant producing a swimming gait in the frequency range of forward terrestrial stepping, i.e. with drives under the limb saturation threshold, so limb oscillators had to be silenced artificially.
