# Supplementary material for: Reproducing Five Motor Behaviors in a Salamander Robot With Virtual Muscles and a Distributed CPG Controller Regulated by Drive Signals and Proprioceptive Feedback
Source: Front Neurorobot. 2020 Dec 23;14:604426. doi: 10.3389/fnbot.2020.604426 (PMC7786271; doi:10.3389/fnbot.2020.604426)
Supplement: Supplementary file 10 [file Data_Sheet_1.pdf]

Supplementary Material

1 SUPPLEMENTARY DATA

Derivation of axial proprioceptive feedback terms in polar coordinates

The axial proprioceptive feedback signal s_i is proportional to the periodic oscillations of the i-th joint angle. This corresponds to a signal in Cartesian coordinates. Our CPG oscillator equations however are written in polar coordinates. To solve this problem, we first rewrite our CPG oscillator in Cartesian coordinates, then add s_i to a Cartesian state derivative, then convert the result back to polar form.

We first note that for any oscillator, the polar transformation $x = r\cos\theta$, $y = r\sin\theta$ implies

$$\begin{aligned} \dot{x} &= \dot{r}\cos\theta - r\dot{\theta}\sin\theta = \frac{\dot{r}}{r}x - y\dot{\theta} \\ \dot{y} &= \dot{r}\sin\theta + r\dot{\theta}\cos\theta = \frac{\dot{r}}{r}y + x\dot{\theta}. \end{aligned} \tag{S1}$$

We can replace $\dot{\theta}$ and $\dot{r}$ using the definition of our isolated oscillator in polar coordinates:

$$\begin{aligned} \dot{\theta} &= \omega \\ \dot{r} &= a(R - r). \end{aligned}$$

We find

$$\begin{aligned} \dot{x} &= \frac{a(R-r)}{r}x - y\omega \\ \dot{y} &= \frac{a(R-r)}{r}y + x\omega, \end{aligned}$$

where $r = \sqrt{x^2 + y^2}$. We choose to couple the feedback signal s to the x state variable:

$$\begin{aligned} \dot{x} &= \frac{a(R-r)}{r}x - y\omega + s \\ \dot{y} &= \frac{a(R-r)}{r}y + x\omega. \end{aligned} \tag{S2}$$

We now have to convert these equations back to polar coordinates. Derivating $r^2 = x^2 + y^2$ with respect to time gives $2r\dot{r} = 2x\dot{x} + 2y\dot{y}$, so

$$\dot{r} = \frac{x\dot{x} + y\dot{y}}{r}.$$

For $\dot{\theta}$, we use a linear combination of (S1), multiplying them by $-y$ and x respectively. This gives $x\dot{y} - \dot{x}y = (x^2 + y^2)\dot{\theta}$, so

$$\dot{\theta} = \frac{x\dot{y} - \dot{x}y}{r^2}.$$

Introducing (S2) into these expressions for $\dot{r}$ and $\dot{\theta}$ gives the polar form of the oscillator with axial proprioceptive feedback:

$$\dot{\theta} = \omega - \frac{s}{r} \sin \theta$$
$$\dot{r} = a(R - r) + s \cos \theta.$$

2 SUPPLEMENTARY FIGURES

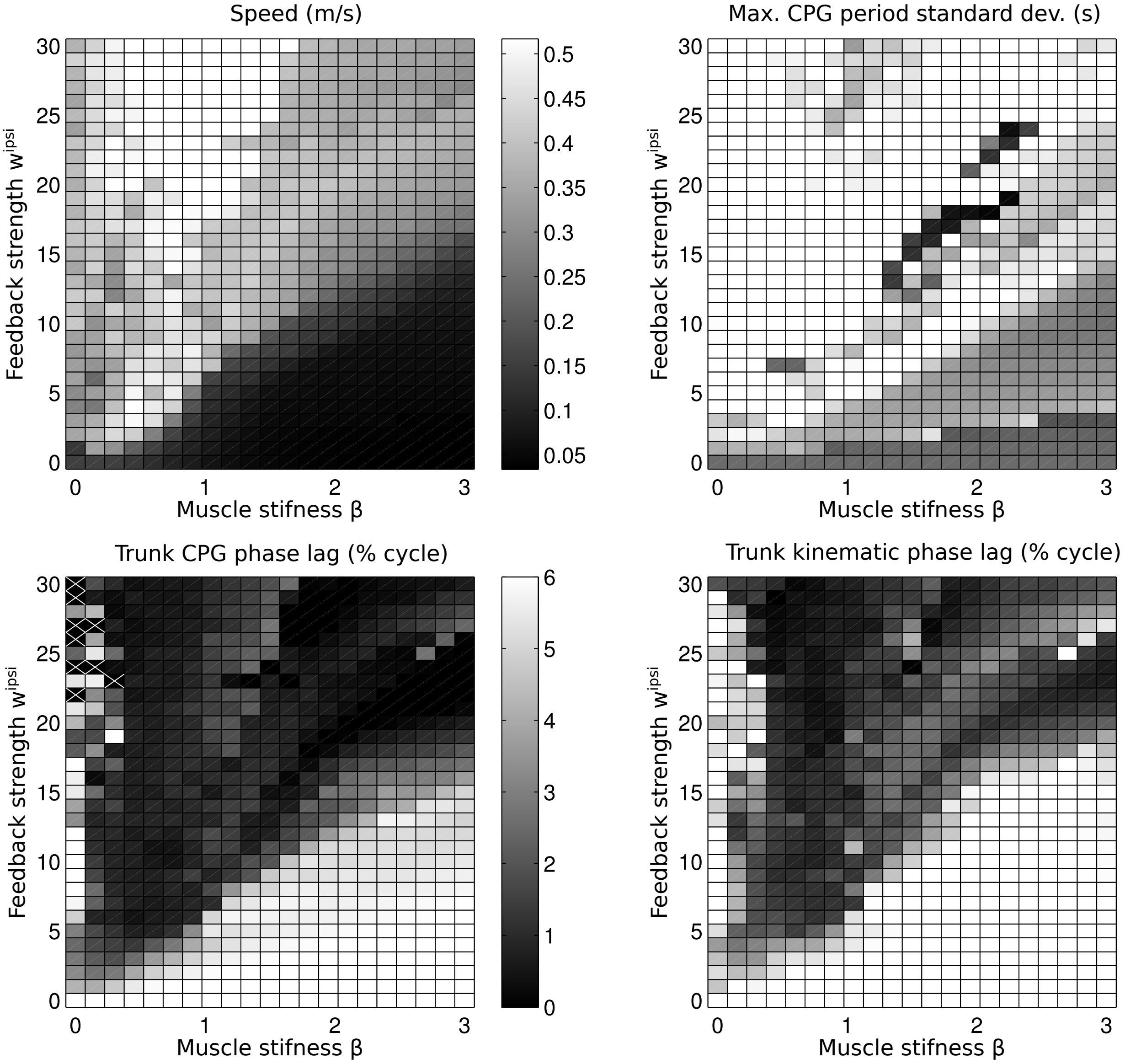

Figure S1. Systematic exploration of the (β, w^{ipsi}) parameter space in swimming simulations for damping $\delta = 0.05$. All tests made with $\alpha = 0.4$, $\gamma = 0$, $w^{\text{contra}} = -w^{\text{ipsi}}$. Upper left and bottom: Without feedback ($w^{\text{ipsi}} = 0$) the CPG produces patterns with phase lags of 6.6%, leading to very small speeds. Increasing the feedback leads to a drop in the CPG and kinematic phase lags (bottom left and bottom right) and an increase in swimming speed. Higher β values, which correspond to stiffer bodies, require stronger feedback for the same effect on the CPG. Upper right: Maximum standard deviation of the cycle period among the oscillators. Only a small region of the parameter space (in black) gives perfectly periodic rhythms.

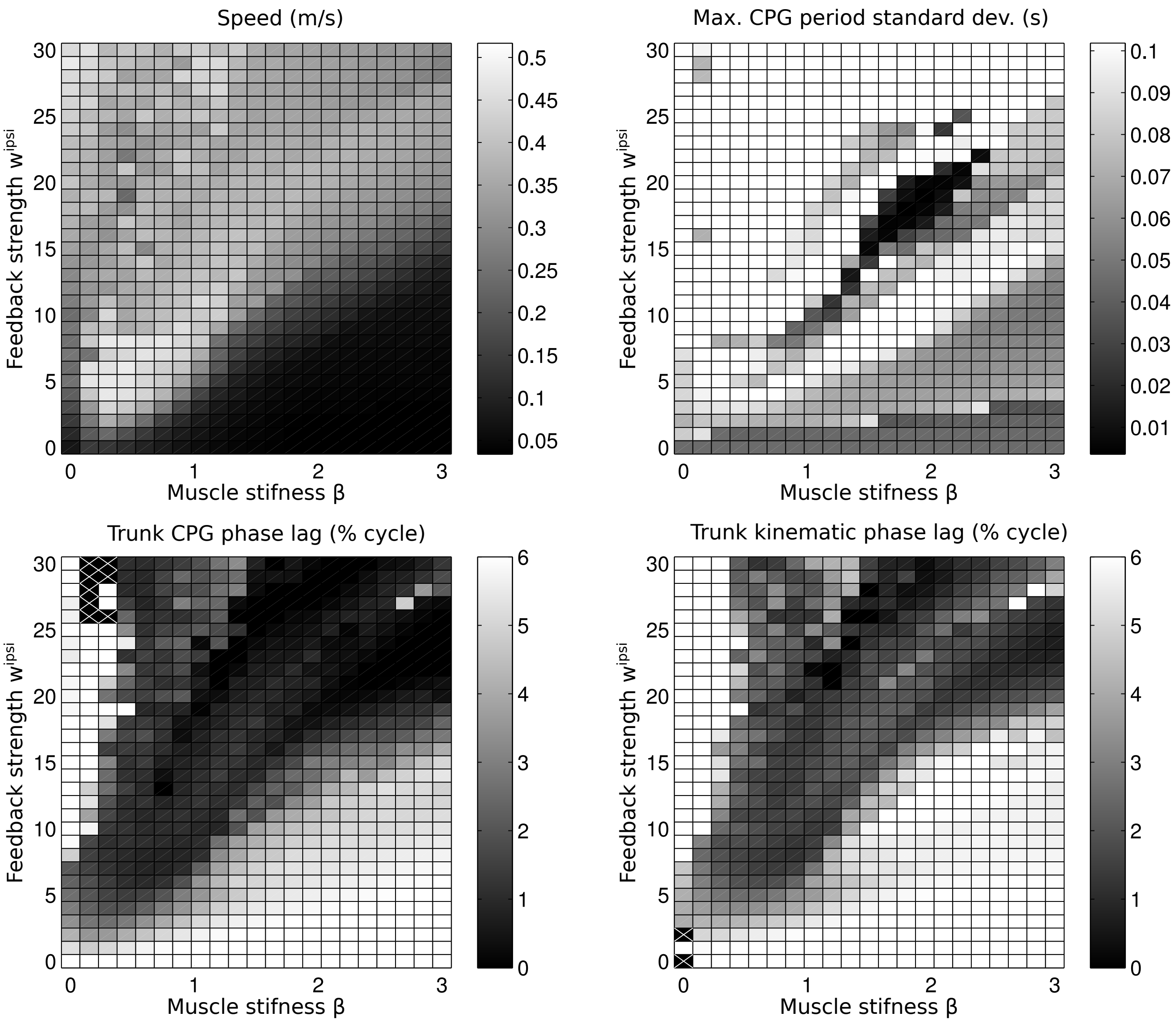

Figure S2. Systematic exploration of the $(\beta, w^{\mathrm{ipsi}})$ parameter space in swimming simulations for damping $\delta = 0.1$. All tests made with $\alpha = 0.4$, $\gamma = 0$, $w^{\mathrm{contra}} = -w^{\mathrm{ipsi}}$. The region with stable CPG activity (upper right, in black) is larger than those in figures S1 and S3.

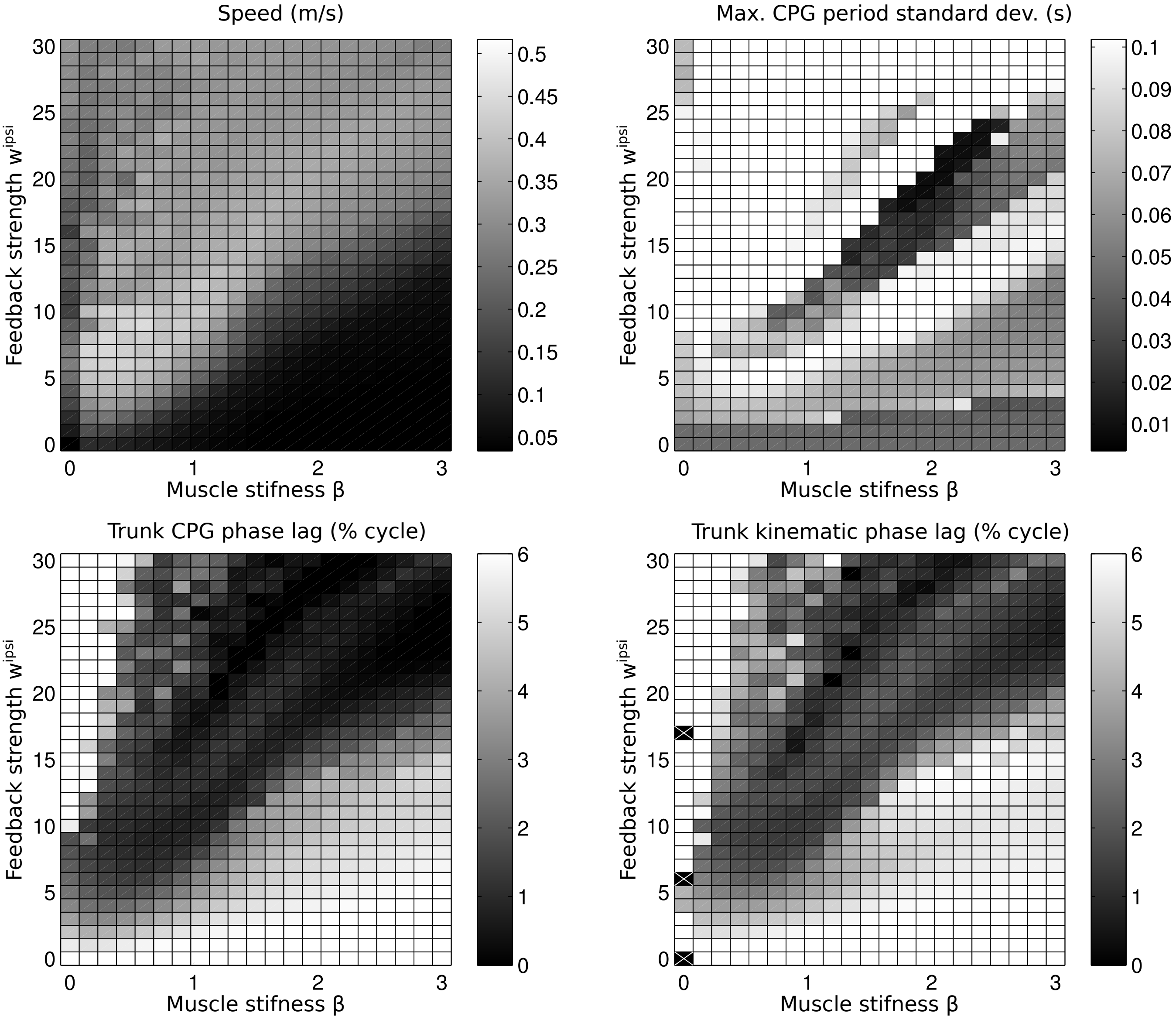

Figure S3. Systematic exploration of the $(\beta, w^{\mathrm{ipsi}})$ parameter space in swimming simulations for damping $\delta = 0.15$. All tests made with $\alpha = 0.4$, $\gamma = 0$, $w^{\mathrm{contra}} = -w^{\mathrm{ipsi}}$.

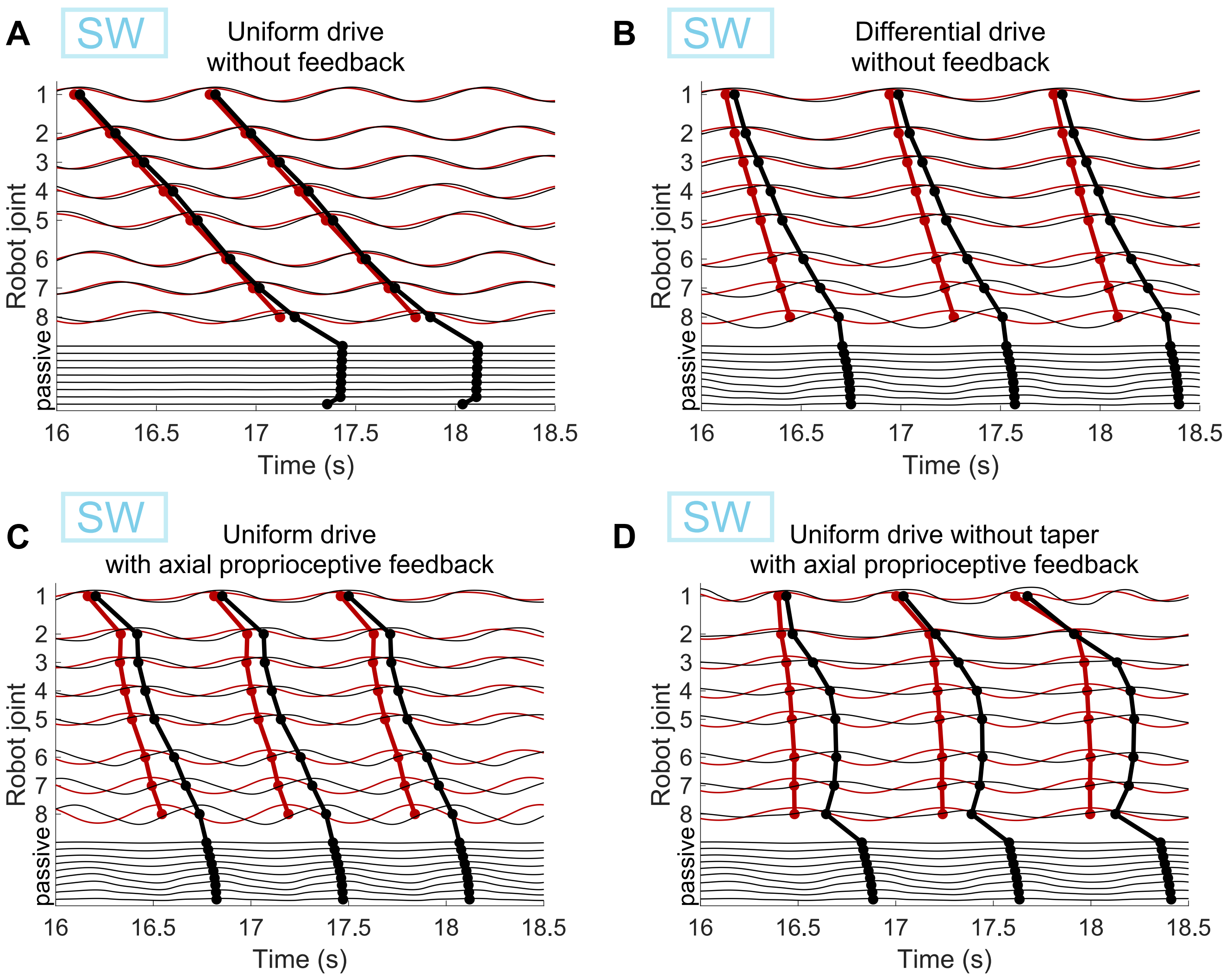

Figure S4. CPG activity and joint kinematics during swimming in simulation. The passive parts at the bottom correspond to the passive joints used to model the caudal fin. **(A)** Without sensory feedback, with uniform drives $d = 1.34$. The CPG produces a traveling wave with large intersegmental phase lag of 6.6%, which fails to travel down the passive fin. **(B)** Without feedback, using a different drive $d = 1.03$ for the first segment gives a more natural phase of about 2%. The kinematic wave follows the rostrocaudal traveling wave of CPG activity, with a delay that increases towards the tail. **(C)** With sensory feedback $w^{\text{ipsi}} = -w^{\text{contra}} = 21$ for all axial oscillators (including in the neck), and a uniform drive $d = 1.34$. The CPG and kinematic waves are similar to the case with differential drives, except for an irregularity near the neck joint. **(D)** With sensory feedback $w^{\text{ipsi}} = -w^{\text{contra}} = 21$, uniform drive $d = 1.34$ and no tapering of muscles in the tail. The model fails to produce a traveling wave.

3 SUPPLEMENTARY MOVIES

Movie S1. *Salamandra robotica II* during swimming (SW) with uniform drive and no feedback. The same drive value is used for all CPG segments and proprioceptive feedback is disabled.

Movie S2. *Salamandra robotica II* during swimming (SW) with differential drive and no feedback. The robot is driven by a differential drive applied to the more rostral oscillators of the central pattern generator model (first segment with drive 0.9, other oscillators drive 1).

Movie S3. *Salamandra robotica II* during forward terrestrial stepping (FTS) with differential drive. The robot is driven by a differential drive applied to the limb vs. axial oscillators of the central pattern generator model (drives of 0.98 and 0.61 respectively).

Movie S4. *Salamandra robotica II* during backward terrestrial stepping (BTS) with differential drive. The robot is driven by a differential drive applied to the limb vs. axial oscillators of the central pattern generator model (drives of 0.44 and 0.23 respectively).

Movie S5. *Salamandra robotica II* during struggling (ST) with differential drive. The robot is driven by a differential drive applied to the limb vs. axial oscillators of the central pattern generator model (drives of 0.38 and 0.27 respectively).

Movie S6. *Salamandra robotica II* during forward underwater stepping with differential drive and active tail segments. The robot is driven by a differential drive applied to the limb vs. axial oscillators of the central pattern generator model (0.69 and 0.42 respectively).

Movie S7. *Salamandra robotica II* during forward underwater stepping (FUS) with differential drive and passive tail segments. The robot is driven by a differential drive applied to the limb vs. trunk oscillators of the central pattern generator model (0.69 and 0.42 respectively), and no drive to the tail segments.

Movie S8. *Salamandra robotica II* during swimming (SW) with uniform drive and phasic axial sensory feedback. The robot is driven by a single drive applied to the whole central pattern generator model (drive 1) and sensory feedback from the axial joints ($w^{\mathrm{ipsi}} = 10$, $w^{\mathrm{contra}} = -10$).

Movie S9. *Salamandra robotica II* splitting in several parts during forward terrestrial stepping. The robot was made to walk with the screws between some modules removed.
